# Structure-Function Relationship of Cytoplasmic and Nuclear IκB Proteins: An In Silico Analysis

**DOI:** 10.1371/journal.pone.0015782

**Published:** 2010-12-23

**Authors:** Balachandran Manavalan, Shaherin Basith, Yong-Min Choi, Gwang Lee, Sangdun Choi

**Affiliations:** 1 Department of Molecular Science and Technology, Ajou University, Suwon, Republic of Korea; 2 Institute for Medical Sciences, School of Medicine, Ajou University, Republic of Korea; University of South Florida College of Medicine, United States of America

## Abstract

Cytoplasmic IκB proteins are primary regulators that interact with NF-κB subunits in the cytoplasm of unstimulated cells. Upon stimulation, these IκB proteins are rapidly degraded, thus allowing NF-κB to translocate into the nucleus and activate the transcription of genes encoding various immune mediators. Subsequent to translocation, nuclear IκB proteins play an important role in the regulation of NF-κB transcriptional activity by acting either as activators or inhibitors. To date, molecular basis for the binding of IκBα, IκBβ and IκBζ along with their partners is known; however, the activation and inhibition mechanism of the remaining IκB (IκBNS, IκBε and Bcl-3) proteins remains elusive. Moreover, even though IκB proteins are structurally similar, it is difficult to determine the exact specificities of IκB proteins towards their respective binding partners. The three-dimensional structures of IκBNS, IκBζ and IκBε were modeled. Subsequently, we used an explicit solvent method to perform detailed molecular dynamic simulations of these proteins along with their known crystal structures (IκBα, IκBβ and Bcl-3) in order to investigate the flexibility of the ankyrin repeat domains (ARDs). Furthermore, the refined models of IκBNS, IκBε and Bcl-3 were used for multiple protein-protein docking studies for the identification of IκBNS-p50/p50, IκBε-p50/p65 and Bcl-3-p50/p50 complexes in order to study the structural basis of their activation and inhibition. The docking experiments revealed that IκBε masked the nuclear localization signal (NLS) of the p50/p65 subunits, thereby preventing its translocation into the nucleus. For the Bcl-3- and IκBNS-p50/p50 complexes, the results show that Bcl-3 mediated transcription through its transactivation domain (TAD) while IκBNS inhibited transcription due to its lack of a TAD, which is consistent with biochemical studies. Additionally, the numbers of identified flexible residues were equal in number among all IκB proteins, although they were not conserved. This could be the primary reason for their binding partner specificities.

## Introduction

NF-κB consists of a family of transcription factors that play central roles in inflammation, immune response, cell proliferation, differentiation and survival [1]. The five members of the mammalian NF-κB transcription factor family are p65 (RelA), RelB, c-Rel, p105/p50 (NF-κB1) and p100/p52 (NF-κB2), which associate with each other to form various transcriptionally active homo- and hetero-dimeric complexes. Each member shares a highly conserved 300 amino acid Rel homology domain (RHD), which mediates DNA binding, dimerization, nuclear localization and association with one of the members of the IκB (inhibitors of NF-κB) protein family. The p65, RelB and c-Rel subunits are positively regulated through transcriptional activation domains (TADs) at their C-terminal ends. Conversely, TADs are absent in p50 and p52; hence, NF-κB is capable of functioning in three different possible ways: by altering κB-site specificity as part of a heterodimer with TAD-containing family members; by repressing transcription as part of a homodimer when bound to κB sites; or by promoting transcription through recruitment of other TAD-containing proteins to κB sites [2]. The IκB protein family is comprised of three functional groups: (a) typical/cytoplasmic IκB proteins, namely IκBα, IκBβ and IκBε, which are present in the cytoplasm of unstimulated cells and undergo stimulus-induced degradation; (b) precursor proteins, p100 and p105, which can be processed to form the NF-κB family members p52 and p50, respectively; and (c) atypical/nuclear IκB proteins, namely IκBζ, Bcl-3 and IκBNS, which are not generally expressed in unstimulated cells but are induced upon activation to mediate their effects in the nucleus [3]. Their classification as “nuclear IκB” is due to the presence of ANK repeats and their localization within the nucleus when expressed in cells [4].

In most resting cells, NF-κB dimers associate with one of the typical IκB proteins such as IκBα, IκBβ and IκBε. These IκB proteins mask the NLS of NF-κB, thereby preventing its translocation into the nucleus. The activation of cells with appropriate stimuli, particularly Toll-like receptor (TLR) ligands or various host immune mediators such as proinflammatory cytokines, including tumor necrosis factor (TNF)-α and interleukin (IL)-1 superfamily proteins, leads to the phosphorylation of cytosolic IκBα and rapid ubiquitin-proteasomal degradation, resulting in the release of NF-κB dimers. These liberated dimers then translocate into the nucleus and bind to the promoter/enhancer regions of target genes, resulting in the regulation of transcription via recruitment of co-activators and co-repressors. The activation of transcription leads to the expression of primary/early response genes, which include three atypical members, IκBζ, Bcl-3 and IκBNS, that play vital roles in the regulation of the transcriptional activity of secondary response genes by acting as either activators or inhibitors in the nucleus [5].

All IκB proteins are characterized by the presence of six to seven ankyrin repeat [6] motifs, which mediate interaction with the RHD of NF-κB dimers. The ANK repeat is roughly composed of a 33-amino acid consensus sequence that appears in multiple copies of numerous proteins [7]. These motifs are known to play an important role in protein-protein interactions while lacking any enzymatic activity [8]. These types of structural motifs are involved in various biological functions such as transcriptional regulation, cytoskeletal organization, cell cycle, cell development and differentiation [9], . In addition to the ANK repeat motif, cytoplasmic and nuclear IκB proteins differ at their N- and C-terminal regions. The amino terminal region of cytoplasmic IκB consists of conserved Ser and Lys residues that undergo phosphorylation and subsequent rapid ubiquitin-proteasomal degradation. Moreover, the carboxyl terminal of cytoplasmic ARD is rich in proline, glutamic acid, serine and threonine (PEST) residues, and this acidic PEST motif has been shown to be indispensable for interactions with NF-κB dimer and its subsequent removal from DNA [11], [12], [13]. Unlike cytoplasmic IκB, nuclear IκB proteins lack both amino-terminal signal-dependent phosphorylation sites and carboxyl terminal PEST regions.

Generally, individual IκB proteins are thought to preferentially associate with a particular subset of NF-κB dimers, although there is little experimental evidence. Cytoplasmic IκBα, IκBβ and IκBε associate with p50/p65, p65/p65 and c-Rel/p65 or p50/p65 dimers, respectively [13], [14], [15], [16], [17]. On the other hand, nuclear Bcl-3 and IκBNS preferentially associate with p50 and p52 homodimers [18], [19], [20]. Among these IκB proteins, nuclear IκBζ has been shown to associate with NF-κB proteins (p50/p50, p50/p65) as well as with other nuclear proteins such as STAT3, p50/p65-CEBP, Brg1 and CEBP1 [21], [22], [23], [24], [25], [26], which regulates its function. All IκB proteins are structurally similar, although the factors governing their diverse functions remain elusive. The solved X-ray crystal structures of IκBα-p50/p65 (PDB ID: 1IKN) and IκBβ-p65/p65 (PDB ID: 1K3Z), and recent molecular modeling studies on the IκBζ-p50/p50 and IκBζ-p50/p65 complexes have revealed the molecular mechanisms of IκB proteins in innate immunity [14], [16], [21]. However, no structural information on the activation and inhibition of the remaining IκB (IκBNS, IκBε and Bcl-3) is known. Based on the biological importance of IκB, we focused on creating models of IκBNS, IκBζ and IκBε to understand how they interact with their partners as well as the molecular basis of its regulation. Furthermore, all modeled structures (IκBNS, IκBζ and IκBε) along with the three crystal structures (IκBα, IκBβ and Bcl-3) were subjected to MD simulation using explicit solvent in order to identify and quantify the differences in flexibility between their respective ARD domains. The results from this study enabled us to glean and understand several key biological insights into the structure-function relationship within IκB proteins.

## Materials and Methods

### Homology modeling

The amino acid sequences of mouse IκBζ, IκBε and IκBNS were retrieved from the NCBI (accession number: NP_085115, AAB97517 and AAL79957) database [27]. Homology modeling was performed using the MOE program (MOE 2008.10; Chemical Computing Group, Ryoka systems Inc, Japan). The modeling procedure has been previously described [21], and the same procedure was followed for modeling of IκBNS and IκBζ. Homology models for IκBNS and IκBζ were built based on the crystal structure of nuclear Bcl-3 protein (PDB ID: 1K1A), which shared highest sequence identities with the two target sequences (36.4% with IκBNS and 37.28% with IκBζ). MOE-Align was used to create a target-template alignment and were manually adjusted based on the alignment results from the multiple sequence alignment of IκBζ ARD reported previously [28]. A series of 10 models for IκBζ and IκBNS were independently constructed by MOE using a Boltzmann-weighted randomized procedure combined with logic for proper handling of the sequence insertions and deletions. There was no difference in the number of secondary structural elements and no significant main chain root mean square (RMS) deviations among the 10 models. However, the model with the best MOE packing score (-2.8597 and -2.4597, respectively) was selected for full energy minimization.

In the case of IκBε, we first used the top two ranked templates (Bcl-3 (1K1A) and IκBβ (1K3Z)) whose sequence identities were 42% and 43.89%, respectively, for model building. With MOE, it is not possible to perform modelling with multiple templates; hence, we opted for Modeller 9V4 [29]. The multiple sequence alignment (MSA) of IκBε with the Bcl-3 and IκBβ sequences was performed using MUSCLE [30]. Three dimensional (3D) models were built based on a distance restraint algorithm imposed by the MSA of the target sequence with template structures by applying the CHARMM force field [31]. An optimization method involving conjugate gradients and MD simulation with simulated annealing was employed to minimize violations of spatial restraints. For the model building, default parameters included in the “automodel” class were used. A series of 20 models were built, from which the best final model was selected based on stereochemical and energetic evaluations.

### Molecular dynamic simulation

All MD simulations were performed using YASARA dynamics with an AMBER99 force field [32]. In this study, we conducted MD simulations for six proteins (IκBα, IκBβ, IκBε, Bcl-3, IκBζ and IκBNS). It is noteworthy that the crystal structures of both IκBα (95–101) and IκBβ (154–157) had the missing residues, which were built prior to the dynamics. A simulation cell was constructed around the model with a 7.9 Å cutoff for Lennard-Jones forces and the direct space portion of electrostatic forces, which were calculated using the Particle Mesh Ewald method. The pKa values of the ionizable groups in the model were predicted and used to assign the protonation states based on pH 7.0. The cell was filled with water, and the AMBER99 electrostatic potential was evaluated for all water molecules; the one with the lowest and highest potential were turned into sodium and chloride counter ions until the cell was neutral. A short steepest descent minimization of all atoms was performed to remove severe bumps in the protein. A start-up simulation was then carried out for 5 picoseconds (ps) using a multiple time step of 1 femtosecond (fs) [33] for intramolecular forces and 2 fs for intermolecular forces, with all heavy protein atoms fixed such that the solvent molecules smoothly covered the protein surface. Simulated annealing minimizations were carried out at 298 K, and the velocities were scaled down every 10 steps for a total time of 5 ps in 500 steps. All systems were equilibrated for 2 ns. Finally, production was carried out for 15 ns by storing the coordinates of all the atoms every 5 ps for further analysis. The simulations were carried out using the AMBER99 force field at 298K and 0.9% NaCl [34]. The final snapshot at the end of the simulation was used as a reference to calculate the root mean square deviation (RMSD) for each amino acid during the last 5 ns MD trajectory. The RMSD calculations obtained from the MD simulations for each case were conducted only after the protein had reached an equilibrium stable state.

### Assessment of the models

The quality of protein geometry was checked by employing ProQ [33], ModFOLD [35] and MetaMQAP [36]. The structural superimpositions and molecular electrostatics involved in the structural analysis were carried out using Superpose v1.0 [37] and Pymol (http://apbs.sourceforge.net), respectively [38].

### Protein-protein docking and binding site prediction

We used unrestrained pairwise docking for the three IκB-NF-κB complexes: IκBε-p50/p65, IκBNS- and Bcl-3-p50/p50 dimer. The individual structures (IκBε, IκBNS and Bcl-3) for docking were obtained from the final snapshots of MD simulation. Prior to docking, only the PDB coordinates (p50 homodimer (PDB ID: 1NFK) and p50/p65 heterodimer (PDB ID: 1NFI)) were pre-processed for energy minimization using the AMBER99 force field distributed in the MOE. Molecular docking was performed using the protein-protein docking software GRAMM-X [39] and ZDOCK [40], which are the most widely used rigid-body protein-protein docking programs for predicting and assessing the interactions between the above complexes. These two programs rank the 100 most probable predictions out of thousands of candidates based on the geometry, hydrophobicity and electrostatic complementarity of the molecular surface. The final docked complexes were chosen from these top 100 lists by considering the knowledge from the biochemical data: the N-terminal RHD of the p50/p50 homodimer is already in a bound state with the DNA (demonstrating the impossibility of binding IκB proteins) [41], [42]. Since only the C-terminal dimerization region remained, we have hypothesized that this region might play a crucial role in the binding of IκB proteins. Our hypothesis is bolstered by previous data on the prediction of the Bcl3-p50/p50 and IκBζ-p50/p50 complexes [4], [21]. Additionally, cytoplasmic IκBε proteins mask the NLS of the p50/p65 complex, thereby preventing its translocation into the nucleus, and this is in agreement with previously solved cytoplasmic IκB proteins [13], [14], [16]. The final docked complexes were subjected to energy minimization in three rounds. Partial charges were assigned to the protein after adding each of the hydrogen atoms (residues of Asp, Glu, Lys and Arg were considered ionized, whereas His residues were considered to be neutral) by employing the AMBER99 force field distributed in the MOE package. In the first round, constraints were applied to the heavy atoms, thereby allowing the mobility of all hydrogen atoms. In the second energy minimization round, only the backbone chain was constrained, whereas the side chains were allowed to move. In the third energy minimization round, only the Cα atoms were constrained, and all other atoms were allowed to move. All of the above energy minimizations were conducted using both the steepest descent and conjugate gradient protocols. The buried surface interaction areas of the dimer models were calculated using the PROTORP server (protein-protein interface analysis) [43].

## Results and Discussion

### Homology models of IκBζ, IκBε and IκBNS

**Figure 1:**
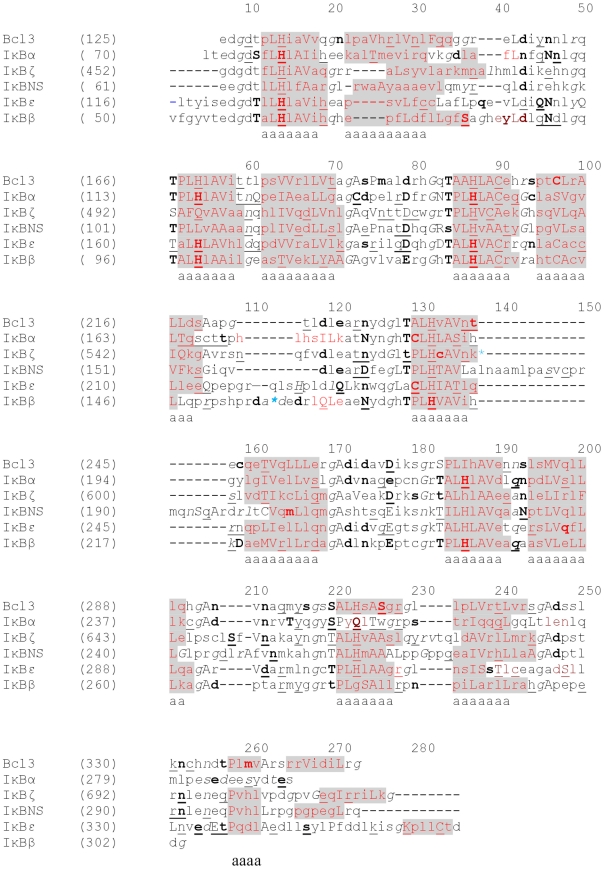
Structure-based sequence alignments of ARD domains. The JOY program was used to annotate the alignments for Bcl-3, IκBα, IκBζ, IκBNS, IκBε and IκBβ. Numbers on top of amino acid sequences are alignment positions. Key to JOY annotations is as follows: solvent inaccessible - UPPER CASE; solvent accessible - lower case; α-helix - dark grey shaded; hydrogen bond to main chain amide - **bold**; hydrogen bond to main chain carbonyl - underline; positive ϕ - italic. The blue colored asterisk represents insertion at that point which has been deleted.

**Figure 2:**
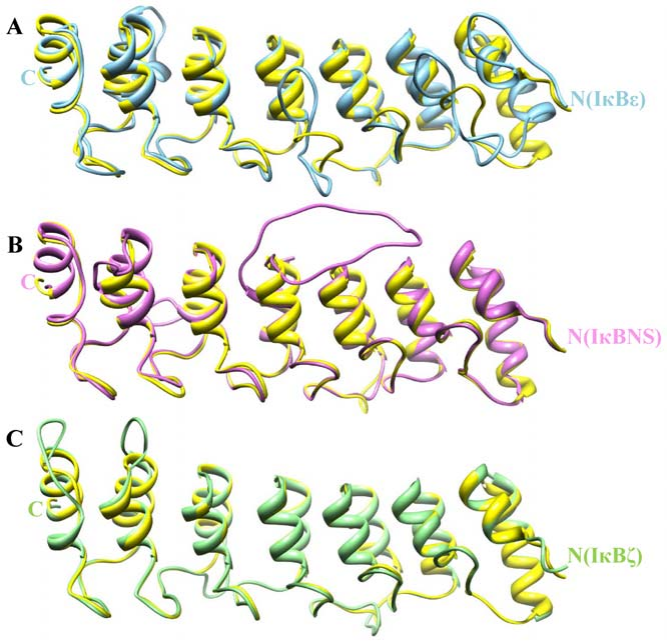
Comparative modeling of ARD. Pair-wise structural superimposition of the modeled ANK repeats: (A) IκBε (colored in sky blue), (B) IκBNS (colored in orchid) and (C) IκBζ (colored in light green) with common structural template Bcl-3 (colored in yellow).

In general, the success of a homology model is related to the degree of sequence identity, the similarity between the target and template, selection of a suitable template and optimal alignment. To date, the X-ray crystal structures of three IκB proteins (IκBα, IκBβ and Bcl-3) have been determined [4], [13], [14]. Based on its high sequence identity, nuclear Bcl-3 acts as a suitable template for IκBζ and IκBNS modelling. In the case of IκBε, IκBβ and Bcl-3 serve as templates. In IκBζ modelling, we deleted a 28 residue insertion, which was located between α1 and α2 of the fourth ANK repeat. The reason for deletion of this insertion region has been previously described [21]. Nevertheless, we also observed a 20 amino acid residues insertion in IκBNS corresponding to IκBζ. In our modelling studies on IκBNS, we did not delete these 20 residues because we wanted to investigate whether or not this portion would reveal any functional significance by MD simulation studies. The sequence alignments, which were used in the construction of the models, are shown in Figure 1. The final models were composed of seven ANK repeats, all of which are in agreement with the secondary structure prediction made by Jpred [44]. The JOY [45] output also shows that the residues in the models are in environments similar to those of the templates. Each ANK repeat of the constructed models depicted two anti-parallel α-helices, followed by a loop of variable length at a right angle. Each repeat began and ended with short β-hairpin turns that protruded away from α-helix. This non-globular fold was stabilized through intra- and inter-repeat hydrophobic interactions. A direct comparison of the modelled structures against the Bcl-3 template revealed the following differences: (i) within the IκBε ANK1 interhelical turn and also in between ANK repeats 1–2, 4–5 and 6–7. Moreover, the α2 helix of ANK6 was larger than the template with an overall RMSD of 2.4 Å. (ii) within the IκBζ ANK6 and 7 interhelical turn and also in between ANK repeats 3–4 and 5–6 with a RMSD of 1.44 Å. (iii) within the IκBNS ANK3 and 7 interhelical turn and also in between ANK3–4 and 5–6 with a RMSD of 1.64 Å (Figure 2). Finally, among the IκB proteins, IκBα and IκBβ have only six ANK repeats, whereas the rest of the proteins (IκBε, Bcl-3, IκBζ and IκBNS) have seven ANK repeats.

### Comparison of cytoplasmic and nuclear IκB proteins

**Figure 3:**
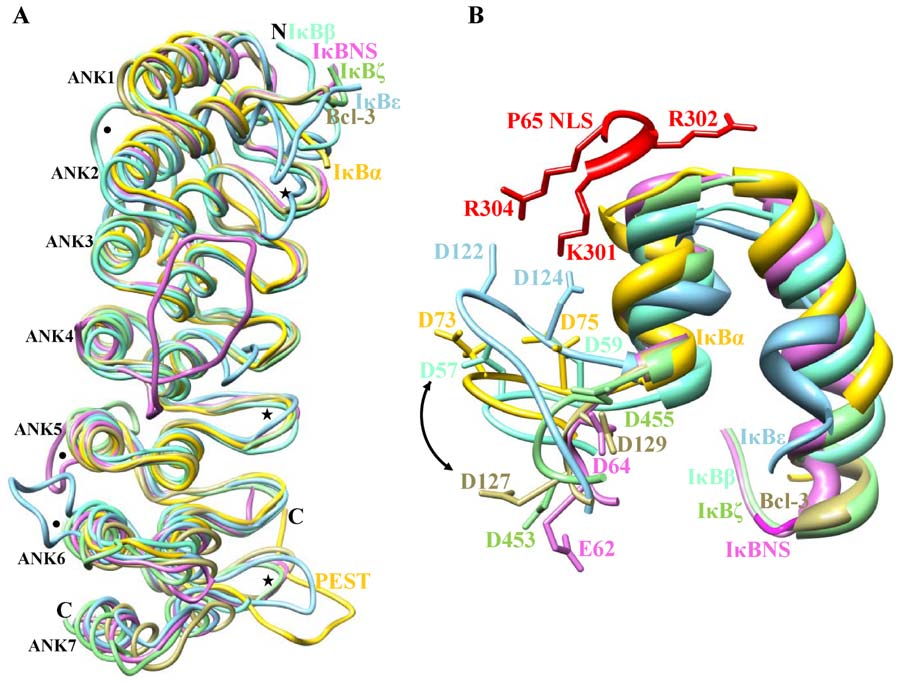
Comparison of IκB structures. (A) Superimpositions of IκBα, IκBβ IκBε, Bcl-3, IκBNS and IκBζ are shown in yellow, aquamarine, sky blue, khaki, orchid and light green, respectively. Major differences were observed within the residue-joining ANK repeats and also between ANK repeats that are represented by dots and asterisks, respectively. (B) Difference between cytoplasmic and nuclear IκB proteins. The conformational differences in the N-terminal residues are indicated by double-headed arrow.

The crystal structures (IκBα, IκBβ and Bcl-3) were superimposed with the modeled structures (IκBε, IκBζ and IκBNS). The results showed that the RMSD ranged from 1.6 to 2.6 Å, indicating that all structures had similar fold (Figure 3A). Significant deviations were observed between the interhelical turn and also in between the ANK repeats. Notably, a long insertion region was observed within the ANK4 repeats of IκBNS. MD simulation studies further illustrated that this portion possesses only two flexible residues (Met182 and Leu183), which allows it to bind with its partners. However, these two residues did not participate in our currently predicted docking poses. Previous studies on IκBζ have shown a similar kind of insertion, but no functional significance was observed regarding binding with its partners [21]. Unlike nuclear protein, cytoplasmic IκBβ contains a unique insertion between ANK3-ANK4, and this region is important for masking the NLS of p65 subunit B [16].

Comparison of the structure of IκBα in its bound state with those of other IκB proteins revealed some significant differences at both the N- and C-terminal ends. For IκBα, N-terminal amino acids 71–76 adopted a hairpin conformation. Acidic residues (Asp73 and Asp75) of β-hairpin loop interacted with basic residues (Lys301, Arg302 and Arg304), which are known to be important for NLS. Other cytoplasmic proteins such as IκBβ (Asp57 and Asp59) and IκBε (Asp122 and Asp124) adopted a similar conformation as that of IκBα, possibly due to interaction with NLS basic residues. On the other hand, the N-terminal region of nuclear IκB followed a perpendicular course in which the corresponding Bcl-3 (Asp126 and Asp128), IκBζ (Asp453 and Asp455) and IκBNS (Glu62 and Asp64) side chains point in opposite directions (Figure 3B). *Huxford et al.,* have shown that p65 NLS is the principal specificity motif involved in determining the binding of cytoplasmic IκB proteins [46]. Conversely, our findings clearly demonstrated that nuclear IκB did not have any influence on p65 NLS, and hence it was not able to bind with p65 containing NF-κB dimer, which is in accordance with previously reported biochemical studies [19], [28]. At the C-terminal end, due to the lack of PEST motif, we eliminated IκBβ and considered only the remaining proteins. The results show that ANK7 of Bcl-3, IκBζ, IκBNS, IκBε and the PEST motif of IκBα were composed of approximately the same number of residues. In addition, these regions occupied similar length and the entire assembly was positioned below ANK repeat 6. However, Bcl-3, IκBζ, IκBNS and IκBε were oriented in the opposite direction of the PEST backbone of IκBα and ended abruptly, leaving the C-termini of the other IκB proteins (Bcl-3, IκBζ, IκBNS, IκBε) as well as IκBα oriented in the opposite direction of the ARD (Figure 3A).

Electrostatic potential studies revealed that the C-terminal regions of cytoplasmic IκB were highly negatively charged (Figure S1A, B and C), and these portions were located near the DNA binding domain. Due to the overall negative charge of DNA, there exists electrostatic repulsion between the complex and the DNA that forces the complex back to the cytoplasm. However, the nuclear protein has both negatively and positively charged surfaces (Figure S1D, E and F) whose functions (positive and negative regulation) depend mainly upon its binding orientation.

### Structure refinement and stability evaluation

**Figure 4:**
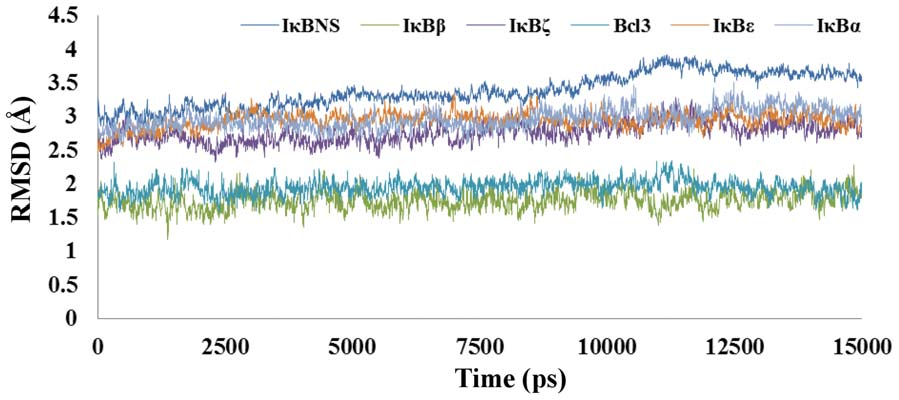
Molecular dynamic trajectory-based analyses of model refinement. RMSD of the Cα atoms with respect to their initial structure show the stable nature of the model after the initial equilibration time.

The available IκB crystal coordinates (IκBα, IκBβ and Bcl-3) along with constructed models (IκBε, IκBζ and IκBNS) were subjected to MD simulation in order to assess the stability of the model. Figure 4 shows the backbone RMSD plot for the protein Cα-atoms with reference to the initial structure and as a function of time. The plot shows that the equilibrium state was reached only after 1 ns of simulation and was kept constant until the end of the dynamics. Superimposition of initial structure with the final refined structure in each case revealed the following structural rearrangements: (i) in between all ANK repeats of IκBα and also in the PEST motif with a RMSD of 3.2 Å, (ii) within the interhelical turn of IκBβ ANK1 and 6 and also in between ANK repeats 1–2, 2–3 and 3–4 with a RMSD of 1.7 Å, (iii) in between all ANK repeats of IκBε with a RMSD of 3 Å, (iv) in between the Bcl-3 ANK repeats of 1–2, 2–3 and 3–4 with a RMSD of 2 Å, (v) within the interhelical turn of IκBNS ANK4 insertion and ANK6 and also in between ANK repeats 1–2, 3–4, 5–6 and 6–7 with a RMSD of 3.5 Å, and (vi) within the interhelical turn of IκBζ ANK1, 6 and 7 and also in between ANK repeats 1–2, 2–3, 3–4, 5–6 and 6–7 with a RMSD of 2.6 Å (Figure S2).

We then took the final snapshots of IκBε, Bcl-3 and IκBNS and subjected them to energy minimization. Model evaluation involved analysis of geometry, stereochemistry and energy distribution of the optimized models. The evaluation listed in Table 1 indicated high quality for all of the models in terms of overall packing. These models were subsequently used for protein-protein docking studies.

**Table 1:** Model evaluation.

	ProQ_LG/MX	ModFOLD_Q/P	MetaMQAP_GDT/RMSD
**IκBα**	6.381/0.235	0.9714/0.0411	63.801/2.62
**IκBβ**	6.795/0.373	0.9880/0.0411	76.705/1.81
**IκBε**	4.715/0.186	0.8786/0.0494	53.213/3.68
**Bcl-3**	6.432/0.367	0.9962/0.0411	79.162/1.58
**IκBNS**	6.172/0.283	0.8731/0.0511	61.661/2.89
**IκBζ**	5.622/0.294	0.9849/0.0411	72.708/2.06

Note: ProQ_LG: >1.5 fairly good; >2.5 very good; >4 extremely good. ProQ_MX: >0.l fairly good; >0.5 very good; >0.8 extremely good. ModFOLD_Q: >0.5 medium confidence; >0.75 high confidence. ModFOLD_P: <0.05 medium confidence; <0.01 high confidence. MetaMQAP_GDT/RMSD: an ideal model has a GDT score over 59 and a RMSD around 2.0 Å.

### Pairwise docking of IκB-NF-κB complex

The structural interactions between IκBα, IκBβ and IκBζ and their partners have been described previously [13], [14], [16], [21]. However, the structural interactions between Bcl-3, IκBβ and IκBNS and their partners are not yet available. The unavailability of these IκB-NF-κB complexes remains as an obstacle to understand the structural basis of their regulation. Regarding the previously solved crystal structure of Bcl-3, the possible interactions with p50/p50 homodimer have been identified by superimposing Bcl-3 and p50 homodimer onto IκBα and p50/p65, respectively [4]. Using the same procedure for Bcl-3, we identified large steric clashes between these complexes. Hence, we performed protein-protein docking studies to obtain the possible binding mode between Bcl-3, IκBβ and IκBNS and its partners. The goal of the protein-protein docking studies were conducted for two main reasons (i) to understand the structural basis of IκB activation and inhibition mechanism and (ii) to validate whether or not the identified flexible residues from the MD studies correlated well with our current molecular docking studies.

The procedure of protein-protein docking is highly computationally oriented. The reliability of the docking results strongly depends on the quality of the docking methods. In order to verify the prediction confidence of the IκB-NF-κB interaction of both methods (GRAMM-X and ZDOCK), we unrestrainedly inputted IκBα-p50/p65 and IκBβ-p65/p65, for which the heterodimeric crystal structures are known, as test cases [14], [16]. The native dimerization structure of IκBα-p50/p65 and IκBβ-p65/p65 were present in the top 100 solutions of both GRAMM-X and ZDOCK and was ranked first by both GRAMM-X and Z-DOCK (Figure S3A and B). This test highlights the feasibility and reliability of GRAMM-X and Z-DOCK applied in IκB-NF-κB docking; hence, we used them in our subsequent docking calculations.

**Figure 5:**
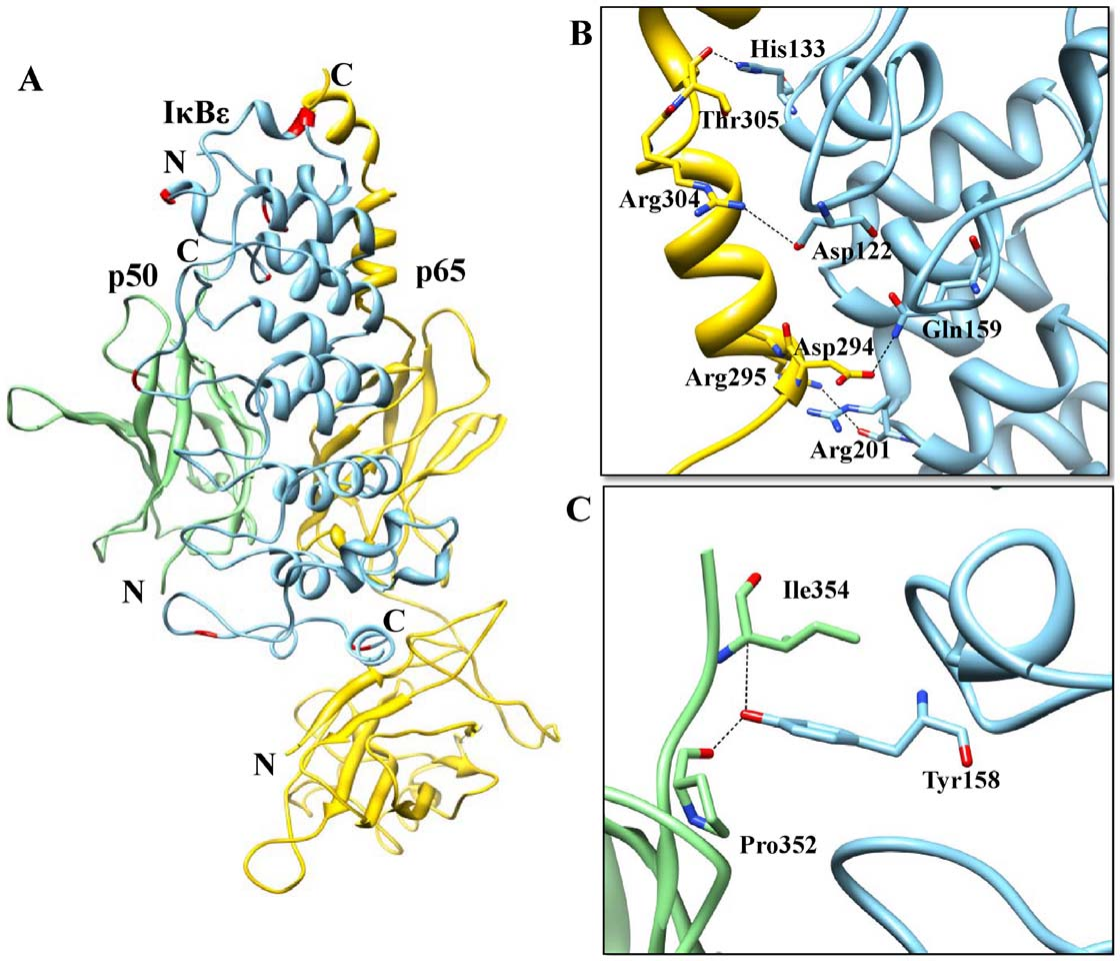
IκBε ARD-p65/p50 heterodimer interface. (A) The p50/p65 heterodimers represented as a ribbon diagram are shown in light green and yellow, respectively. Docked IκBε is represented in sky blue color in the ribbon diagram, and flexible residues involved in the interactions are in red color. (B) p65-IκBε binding interface. Side chains of the amino acids contributing to hydrogen bonding formation (marked as black dotted lines) are represented by a stick model with the residue names and numbers shown next to them. (C) p50-IκBε binding interface is also represented in a similar fashion as (B).

**Figure 6:**
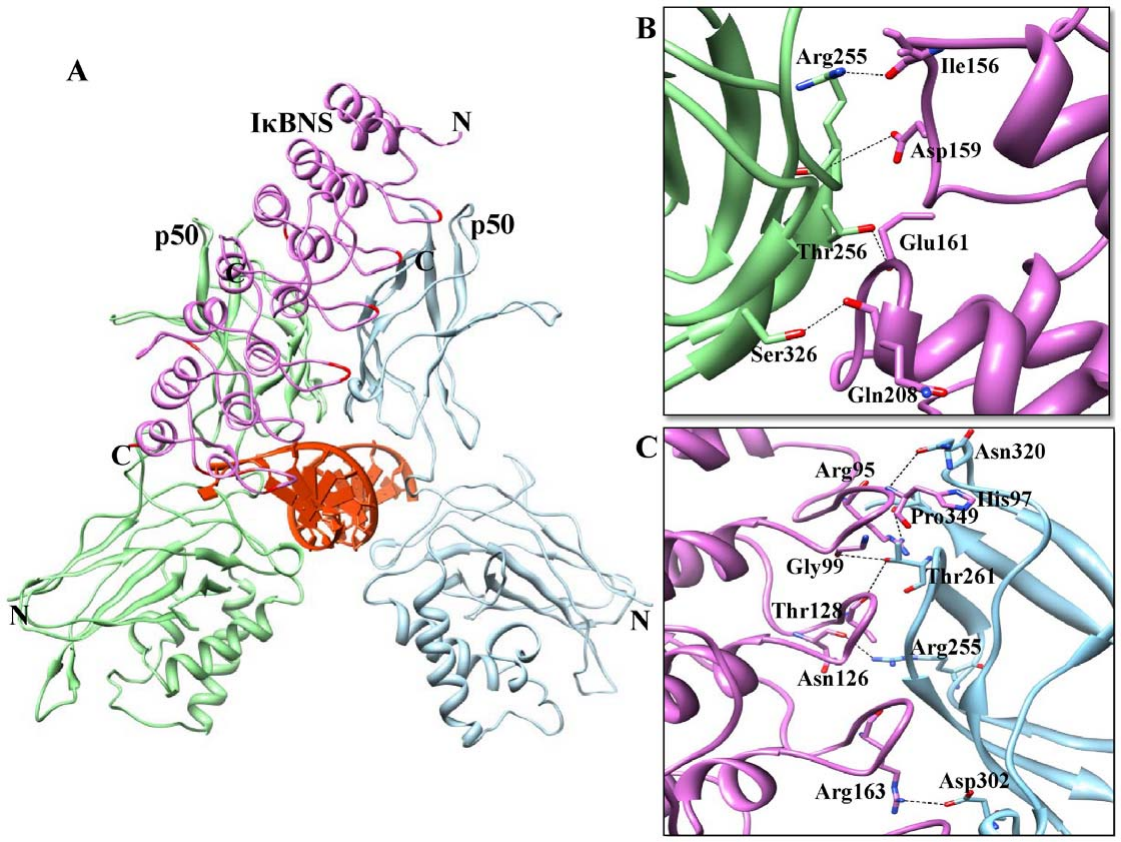
IκBNS ARD-p50/p50 homodimer interface. (A) The p50/p50 homodimers represented as a ribbon diagram are shown in light green and sky blue, respectively. Docked IκBNS is represented in orchid color in the ribbon diagram, and flexible residues involved in the interactions are red colored. (B) The p50 (chain A)-IκBNS binding interface. Side chains of the amino acids contributing to hydrogen bonding formation (marked as black dotted lines) are represented by a stick model with the residue names and numbers shown next to them. (C) The p50 (chain B)-IκBNS binding interface is also represented in a similar fashion as (B).

**Figure 7:**
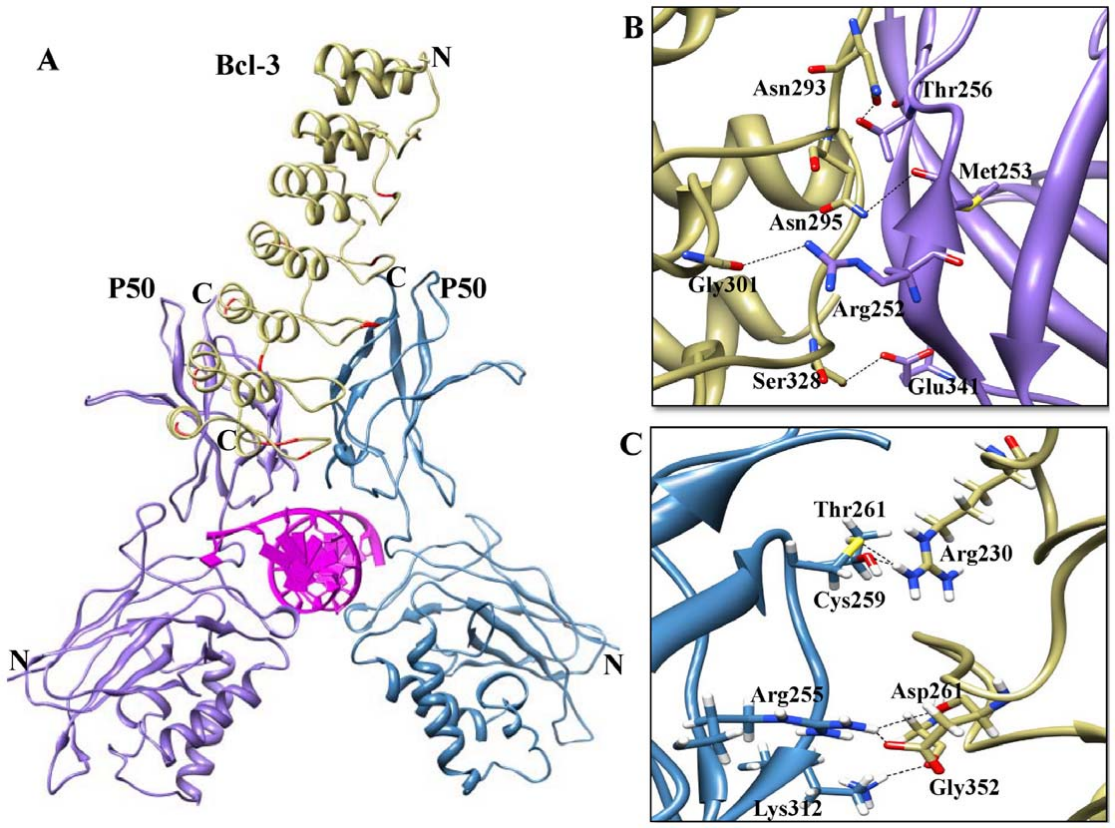
Complex A (Bcl-3 ARD-p50/p50 homodimer) interface. (A) The p50/p50 homodimers represented as a ribbon diagram are shown in purple and blue, respectively. Docked Bcl-3 is represented in khaki color in the ribbon diagram, and flexible residues involved in the interactions are red colored. (B) The p50 (chain A)-Bcl-3 binding interface. Side chains of the amino acids contributing to hydrogen bonding formation (indicated by black dotted lines) are represented by a stick model with the residue names and numbers shown next to them. (C) The p50 (chain B)-Bcl-3 binding interface is also represented in a similar fashion as (B).

**Figure 8:**
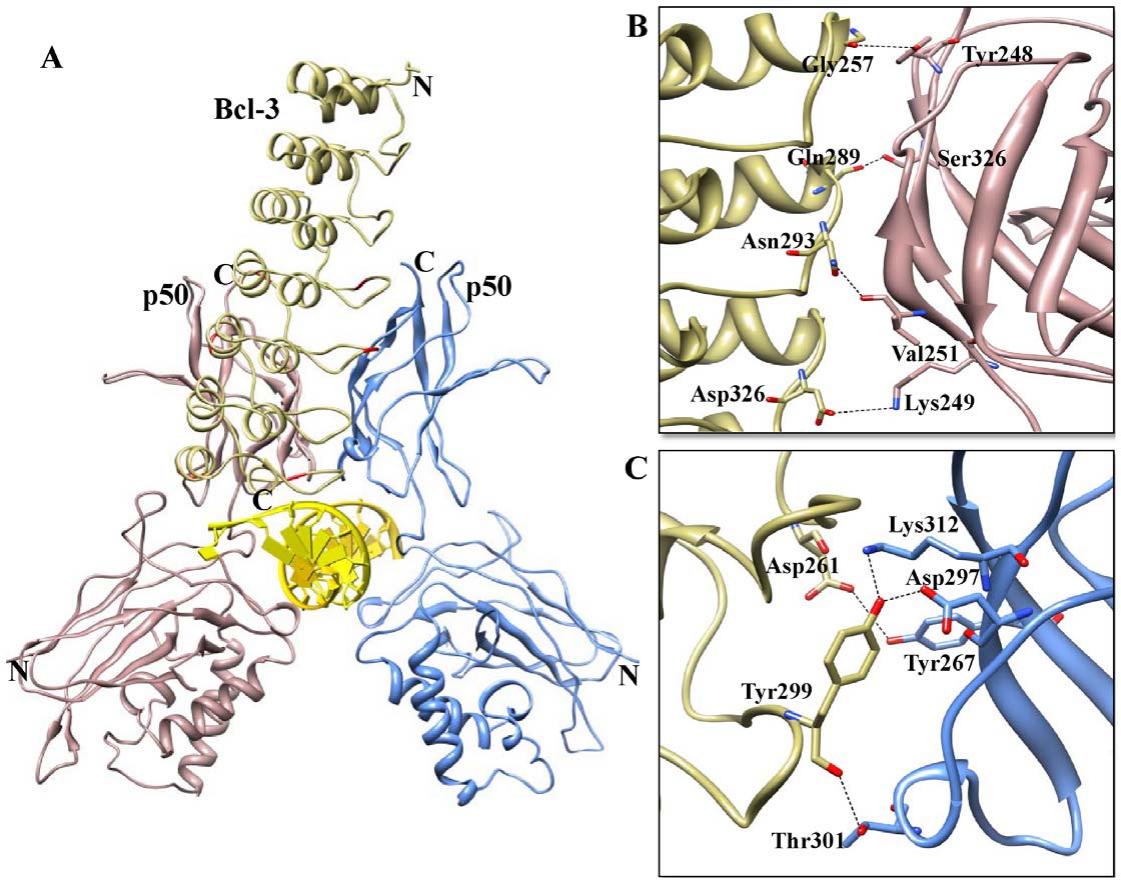
Complex B (Bcl-3 ARD-p50/p50 homodimer) interface. (A) The p50/p50 homodimer represented as a ribbon diagram are shown in rosy brown and blue, respectively. Docked Bcl-3 is represented in khaki color in the ribbon diagram, and flexible residues involved in the interactions are red colored. (B) The p50 (chain A)-Bcl-3 binding interface. Side chains of the amino acids contributing to hydrogen bonding formation (indicated by black dotted lines) are represented by a stick model with the residue names and numbers shown next to them. (C) The p50 (chain B)-Bcl-3 binding interface is also represented in a similar fashion as (B).

To elucidate how IκBε binds with the p50/p65 complexes and prevents their translocation into the nucleus, and also how Bcl-3 and IκBNS bind to the p50 homodimer, which mediates transcriptional activation and inhibition, we conducted unrestrained rigid-body docking of Bcl-3-p50/p50, IκBε-p50/p65 and IκBNS-p50/p50 dimers. Each docking returned the 100 most probable models from unbound monomer components. Thus, each complex received a total of 200 candidate models separated into two sets. Some models from the same set had similar conformations, whereas most differed considerably from one another. There were some shared models (intersection) across both sets for each complex. These shared models were considered as more confident solutions than others. The optimal docking solution for each complex was selected from the 200 candidates based on the following criteria: (i) models that do not exist in the intersection of the two resulting sets were excluded; (ii) include only those shared models in which the binding region is supported by the experimental data (see methods). This two-step filtering method led to a unique solution. The ZDOCK/GRAMM-X ranking and the buried surface interaction area of all optimal models are provided in Table 2. The top-ranked complex from Table 2 is considered as the final complex that was subsequently subjected to energy minimization and the identification of the residual interface. Additionally, in order to compare the subtle differences observed among various multiplexes (IκBε-p50/p65, IκBNS-p50/p50, Bcl3-p50/p50 activation and Bcl3-p50/p50 inhibitory complexes), we have listed the top five complexes in each multiplex whose orientations are more or less similar to the final complexes as shown in Figures 5, 6, 7 and 8. Their corresponding interacting residues, H-bonds, salt bridges and interface areas are presented in Tables S1.

**Table 2:** Ranking and interaction area of the selected docking complex.

	ZDOCK	GRAMM-X	Interaction area (Å^2^)
**IκBε-p50/p65**	4	24	1339
**IκBNS-p50/p50**	2	56	1708
**Bcl-3-p50/p50 (a)**	45	62	1585
**Bcl-3-p50/p50 (b)**	57	38	1246

### IκBε-p50/p65

Previous studies have shown that IκBε inhibits the DNA binding of p50/p65 subunits [2], [15], thereby modulating the transcriptional activity mediated by NF-κB. However, there is no structural interpretation of this experimental data. To this end, we carried out molecular docking studies that led to two candidate models after two-step filtering, which are ranked 4/24 by ZDOCK/GRAMM-X respectively. However, we took the top ranked complex provided by ZDOCK and identified the crucial interface residues. The interface of the IκBε-p50/p65 complex was separated from p50/p65 heterodimer by 1339 Å^2^ and from IκBε by 1360 Å^2^ (Figure 5A). In this complex, we evaluated the interacting residues, number of interchain H-bonds, salt bridges and interface surface area (Table 3). In particular, IκBε and the p50/p65 complexes form seven hydrogen bonds, which included a double H-bond between IκBε Asp122 and p65 Arg304. Further, the OH group of Tyr158 forms two H-bonds with the carbonyl oxygen of Pro352 and backbone nitrogen of Ile354 (Figure 5B and C). Five salt bridges were also observed between the complexes. The interface region of IκBε is composed of three negatively charged and four positively charged residues. In the case of p50-p65 complex interface, four negatively and five positively charged amino acids are exposed. These data suggest that the predominant interactions between IκBε and p50/p65 are based on electrostatic interactions.

**Table 3:** List of interactions between IκBε and the p50/p65 heterodimer.

	Interacting residues
**IκBε**	***Asp122***, **Asp124**, Leu126 Leu129, Ala130, Ile132, ***His133***, Ala135, Ser137, Val138, Asn155, Asn156, Leu157, *Tyr158*, ***Gln159***, Leu164, **His167**, Leu168, **Asp169**, Gln189, **His190**, Asn203, ***Arg209***, Trp232, Thr266, Ile355, Ser356
**P65 subunit A**	**Asp293**, ***Asp294***, ***Arg295***, Ile298, **Glu299**, **Lys301**, **Arg302**, ***Arg304***, ***Thr305***, Phe309, Ile312, Met313
**P50 subunit B**	**Arg258**, Thr325, **Lys326**, Pro327, Pro347, Tyr351, ***Pro352***, **Glu353**, ***Ile354***

Note: The charged residues are in bold while the residues involved in the formation of H-bonds are in italic.

The majority of the specific interactions are made between ANK repeats 1, 2 and 3 of IκBε and the NLS-containing C-terminal portion of p65 subunit A (NLS polypeptide; p65 amino acids 291–319). These interactions are similar to those observed in between the IκBα-p50/p65 and IκBβ-p65/p65 complexes [14], [16]. In addition to the NLS, IκBε uses the loop region in between ANK repeats 3–4 and 4–5 to make contact with the p50/p65 dimerization region. The NLS polypeptide of p65 subunit A possesses two helices with an approximately orthogonal relative orientation. The last four amino acids of the first helix, Lys301, Arg302, Lys303 and Arg304, constitute the functional NLS. Three of these residues, Lys301, Arg302 and Arg303, contact six amino acids (Asp122, Asp124, Ile32, His133 Glu134 and Gln155) from the first ANK repeat of IκBε. All six amino acids are identical between IκBα and IκBβ. Therefore, it is not surprising that the contacts made between p65 NLS and IκBε, IκBα and IκBβ are highly homologous between these complexes. Like IκBα and IκBβ, the NLS polypeptide region of NF-κB p65 subunit might be the key specificity-determining motif for the interaction with IκBε [46].

The present docking pose revealed that IκBε is tightly bound to the NLS of p65 subunit A. However, due to lack of the p50 NLS portion in the crystal structure, we were not able to identify its interaction. It should also be noted that the primary sequences of p50 (40–370) and p65 (29–347) share >80% similarity, with a 32 amino acid insertion at the p50 N-terminal end. Additionally, the p65 NLS (Lys301, Arg302 and Arg304) corresponding residues are Lys360, Arg361 and Lys363, respectively. By assuming that both structures are identical, we have superimposed p65 homodimer (considering p65 subunit B as p50) onto the IκBε-p50/p65 complex, followed by energy minimization of the resulting complex. The results reveal that there are strong interactions between IκBε (Leu116, Thr117, Asp124 and Thr125) and p50 NLS subunit B (Lys301, Arg302 and Arg304). Of the four residues in IκBε, Asp124 and Thr125 are conserved between IκB families (Figure 1). This current docking pose is likely to prevent the complex from entering into the nucleus, which is in agreement with previous biochemical reports [15]. The IκBε function is similar to IκBα, but IκBε degradation and resynthesis occurs with considerably delayed kinetics when compared to IκBα [3]. The stimulus-induced degradation of IκB proteins (which masks the NLS of NF-κB dimer) leads to the translocation of NF-κB into the nucleus followed by binding with the DNA and subsequent regulation of the transcription of numerous target genes. Newly synthesized IκBε translocates into the nucleus and binds to NF-κB. This complex must be exported back to the cytoplasm, thereby terminating the transcriptional process, in order to prevent excessive activation that leads to endotoxin tolerance. In the case of IκBα, its PEST motif (Figure S1A, highlighted in dotted circle) participates in electrostatic repulsion with the phosphate group of DNA, thereby bringing the IκBα-p50/p65 complex back to the cytoplasm [13], [14]. However, no well-defined PEST motif has been reported for IκBε [15]. Moreover, the electrostatic potential studies of IκBε revealed a negatively charged surface present in ANK7 that is composed of Glu342, Asp343, Ser346, Tyr347, Pro349, Asp351, Asp352, Ser356 and Pro359 residues (Figure S1C, highlighted in dotted circle). Our results suggest that this region might play an important role in exporting the complex back to the cytoplasm.

### IκBNS-p50/p50

TLR-dependent gene induction is also regulated by nuclear IκB proteins such as Bcl-3, IκBζ and IκBNS. *In vitro* experiments indicated that IκBNS is induced by IL-10 or LPS and selectively inhibits MyD88-dependent genes, including IL-6, IL-12p40 and IL-8, by association with DNA-bound p50 homodimer [19]. IκBNS is structurally similar to IκBζ, but unlike IκBζ, which regulates transcription by binding with DNA bound p50 homodimer [21], the structural factors responsible for IκBNS-mediated negative regulation of TLR-induced NF-κB-dependent genes remain unknown. To this end, we carried out molecular docking studies and identified the final IκBNS-p50/p50 complex (Figure 6A). The buried surface at the interface of the IκBNS-p50/p50 complex was separated from the p50/p50 homodimer by 1708 Å^2^ and from IκBNS by 1844 Å^2^. We evaluated the interacting residues, the number of interchain H-bonds, salt bridges and the interface surface area (Table 4). Ten hydrogen bonds were present at the interface, of which ten are donated by IκBNS and nine are from the p50/p50 complex (Table 3). These included two hydrogen bonds formed between the OH group of Thr261 (IκBNS) and the carbonyl group of p50 (subunit B) Gly99 and Thr128 (Figure 6B and C). The strongest salt bridge was observed between IκBNS Arg95 and P50 Asp350. The interface region of IκBNS is composed of six negatively charged and nine positively charged residues. In the case of p50/p50 complex, the interface region consists of seven negatively and eight positively charged amino acids. These data suggest that the predominant interaction between IκBε and p50/p50 is based on the electrostatic interaction.

**Table 4:** List of interactions between IκBNS and the p50/p50 homodimer.

	Interacting residues
**IκBNS**	**Arg90**, Gln91, Ile94, ***Arg95***, **Glu96**, ***His97***, ***Gly99***, ***Asn126***, ***Thr128***, **Asp129**, **His130**, Gly132, Phe152, ***Ile156***, Val158, ***Asp159***, Leu160, ***Glu161***, ***Arg163***, **Asp164**, Phe165, Leu207, ***Gln208***, Met209, Gly210, Ser212, Thr214, Ile218, **Lys219**, Ser220, Asn221, **Arg249**, Phe251, **His257**, Gly285, **Asp287**, Pro288, Thr289, **Arg291** Asn295, **Lys302**, Gln313
**P50 subunit A**	**His64**, **Lys74**, Asn75, **Lys77**, **Arg252**, **Asp254**, ***Arg255***, ***Thr256***, **Ala257**, Cys259, ***Ser326*** *,* **Glu341**, **Lys343**, Pro344, Tyr348, Leu346
**P50 subunit B**	***Arg255***, Cys259, ***Thr261***, Gly262, Gly263, **Glu264**, **Glu265**, **Asp297**, **Ser299**, Pro300, Thr301, ***Asp302***, **Lys312**, **Lys315**, ***Asn320*** *,* Tyr348, ***Pro349***, **Glu350**

Note: The charged residues are in bold while the residues involved in the formation of H-bond are in italic.

The majority of the specific interactions are made between IκBNS ANK repeats 2–5 and the dimerization domain of p50/p50. These interactions are similar to those observed between IκBζ and the p50/p50 complex [21]. Additionally, ANK7 interacts with the N-terminal region of p50 subunit B. Although the binding orientations are similar for both IκB proteins, there might be some differences in the regulation of LPS-mediated secondary/late response gene induction by IκBζ and IκBNS. Both proteins are induced upon TLR stimulation; IκBζ binds with DNA-bound p50/p50 homodimer and regulates transcriptional activity through its TAD in the N-terminal non-ARD [23]. Whereas for IκBNS, there is no well-defined TAD, indicating that IκBNS-mediated transcription is unfeasible. Additionally, our docking model places the finger loop region of ANK6 nearby the minor groove region of the DNA. Electrostatic potential studies have shown that the basic patches present at this position make this candidate suitable for polar interactions with the backbone of DNA (Figure S1E, highlighted in circle). Due to the strong electrostatic interactions between the IκBNS-p50/p50 complex and DNA phosphate backbone, the complex remains as such in the nucleus. Our results suggest that this complex might act as an inhibitor for two reasons: (i) due to lack of TAD in both IκBNS and p50 homodimer, and (ii) lack of negatively charged surface in IκBNS, which is located near DNA, making it unfeasible to replace the p50 homodimers with transcriptionally active dimers. Therefore, IκBNS suppresses MyD88-dependent genes by associating with DNA-bound p50 homodimers, thereby preventing the binding of transcriptionally active NF-κB dimers to DNA. Furthermore, in IκBNS-deficient mice, LPS-induced activation of NF-κB is prolonged [19]. As a result, mice turned out to be susceptible to intestinal inflammation caused by the disruption of the epithelial barrier. Briefly, the present docked complex mode clearly explains the inhibitory mechanism of IκBNS, thereby preventing excessive inflammation, which is in accordance with previous biochemical studies [19], [47].

### Bcl-3-p50/p50

*In vitro* studies revealed that overexpression of Bcl-3 results in NF-κB-mediated gene expression or gene suppression, depending on the conditions, via association with p50 or p52 homodimer, which indicates its potential role as either an activator or inhibitor in TLR signaling [5]. Binding of Bcl-3 with p50 or p52 homodimer leads to three different results: (i) p50 and p52 lack TADs and are therefore repressive, but binding to Bcl-3 (which contains a well-defined TAD) possibly confers transcriptional activity, (ii) Bcl-3 might facilitate transcription by displacing the p50 and p52 homodimers, thus allowing transcriptionally active dimers to take their place, and (iii) Bcl-3 inhibits NF-κB-dependent transcription by stabilizing the p50 and p52 homodimers that are bound to the κB sites [2], [4], [20], [48]. Since the structural mechanisms of Bcl-3-mediated activation and inhibition remain elusive, we conducted molecular docking studies and identified two different orientations of Bcl-3 binding with p50 homodimer, thus illustrating the dual roles exhibited by Bcl-3 in NF-κB signaling. We named these complexes as A and B, respectively. In complex A, Bcl-3-p50/p50 mediates transcription via Bcl-3 TAD, whereas in complex B, Bcl-3 facilitates transcription by displacing p50 homodimer, thereby allowing binding of the transcriptionally active dimers.

### Complex A

The buried surface at the interface of the Bcl-3-p50/p50 complex is separated from the p50/p50 homodimer by 1585 Å^2^ and from Bcl-3 by 1644 Å^2^ (Figure 7A). Nine H-bonds are present at the interface, of which seven are donated by Bcl-3 and eight by the p50/p50 complex (Table 5). These included a double hydrogen bond between p50 (subunit B) Arg255 and Bcl-3 Asp261. The Bcl-3 Arg230 side chain also forms two H-bonds with p50 (subunit B) Cys259 and Thr261 (Figure 7B and C). Two salt bridges were also observed between Bcl-3 Asp261 and Asp326 as well as P50 Arg255 and Lys343. The predominant interactions between Bcl-3 and p50/p50 are based on the electrostatic interactions. The majority of the specific interactions are made between Bcl-3 ANK repeats 4–7 and the dimerization domain of p50/p50. No portion of Bcl-3 was observed as overlapping the DNA binding region or in close proximity to the DNA. These interactions are identical to those observed in the IκBζ-p50/p50 complex [21]. When we compared the Bcl-3 binding orientation with the cytoplasmic IκB protein crystal structures, the results showed that Bcl-3 binding tilted 15 degrees towards the right hand side. It is noteworthy that DNA-bound p50/p50 dimer did not mediate transcription due to the lack of TAD. However, previous biochemical studies have shown that Bcl-3 possesses a well-defined TAD [2], [48]. Based on our current model, we propose that Bcl-3 can mediate transcriptional activity by binding with DNA-bound p50/p50 dimer, thereby providing a transactivation domain to the NF-κB complex. Previous studies using a Bcl-3 ^−/−^ macrophage cell line have shown that Bcl-3 positively regulates the expression of IL-10 [20]. Finally, the structural mechanism of Bcl-3 positive regulation can be elucidated by our present docked complex.

**Table 5:** List of interactions between Bcl-3 and the p50/p50 homodimer (Complex A).

	Interacting residues
**Bcl-3**	Met191, **Asp226**, **Glu228**, Ala229, ***Arg230***, **Arg252**, ***Asp261***, Val263, Ile265, Gly268, Leu288, Gln289, **His290**, Gly291, Ala292, ***Asn293***, Val294, ***Asn295***, Gln297, Met298, Tyr299, ***Gly301***, Leu320, Val321, **Arg322**, Ser323, Gly324, Ala325, **Asp326**, ***Ser328***, Leu329, **Lys330**, Asn331, Cys332, Asn334, **Arg351**, ***Gly352***
**P50 subunit A**	**Lys74**, **Lys249**, Ile250, Val251, ***Arg252***, ***Met253***, **Asp254**, **Arg255**, ***Thr256***, Ala257, Pro324, Ser326, Val327, Phe328, ***Glu341***, Pro342, Pro344, Phe345, Leu346, Tyr348
**P50 subunit B**	***Arg255***, ***Cys259*** *, * ***Thr261***, Gly262, Gly263, **Glu264**, **Glu265**, Tyr267, Ser299, Thr301, **Asp302**, Val310, ***Lys312***, **Glu350**

Note: The charged residues are in bold while the residues involved in the formation of H-bond are in italic.

### Complex B

The buried surface at the interface of the Bcl-3-p50/p50 complex is separated from the p50/p50 homodimer by 1246 Å^2^ and from Bcl-3 by 1216 Å^2^ (Figure 8A) Eight H-bonds are present at the interface, of which six are donated by Bcl-3 and eight by the p50/p50 complex (Table 6). These included two hydrogen bonds formed between the OH group of Bcl-3 Tyr299 and the p50 (subunit B) side chains of Lys312 and Asp297. Additionally, the carbonyl oxygen of Bcl-3 Tyr299 forms a H-bond with the OH group of p50 (subunit B) Thr301 (Figure 8B and C). A salt bridge is also observed between Bcl-3 Asp326 and P50 Lys249. In our final model, the interactions are made between Bcl-3 4-6 ARD and the DD of the p50 subunits. Though the binding orientation is similar to complex A and IκBNS-p50/p50, the major difference observed for complex B is the position of Bcl-3 ANK7 placed near the minor groove of the DNA, with four residues (Asp326, Ser327, Ser328 and Lys330) possibly as good candidates for making polar interactions with the DNA backbone (Figure S1D, highlighted in dotted circle). Generally, the ability of Bcl-3 to facilitate or inhibit transcriptional activity is determined by post-translational modification [48], [49], [50]. Though there is no well-defined PEST motif in Bcl-3, we made a note of few Ser residues located at the C-terminal end. During post-translational modification, phosphorylation of these Ser residues might consequently destabilize the Bcl-3-p50/p50-DNA complex by electrostatic repulsion with the DNA phosphate groups. This results in binding of the active NF-κB dimer to the DNA, resulting in transcription. However, such transcriptionally regulated genes have not yet been identified.

**Table 6:** List of interactions between Bcl-3 and the p50/p50 homodimer (Complex B).

	Interacting residues
**Bcl-3**	**Arg230**, Leu254, **Glu255**, **Arg256**, ***Gly257***, **Asp259**, Ile260, ***Asp261***, Val263, **Asp264**, Ile265, **Lys266**, Gly268, Leu288, ***Gln289***, **His290**, Gly291, Ala292, ***Asn293***, ***Tyr299***, Ser300, Gly301, ***Asp326***, **Arg351**, Gly352
**P50 subunit A**	**Glu73**, **Lys74**, ***Tyr248*** *, * ***Lys249***, ***Val251***, Arg252, Met253, **Asp254**, Thr256, Pro324, ***Ser326***, **Lys343**, Pro344, Phe345, Leu346, Tyr348
**P50 subunit B**	Gly263, **Glu264**, **Glu265**, ***Tyr267***, ***Asp297***, Ser299, ***Thr301***, ***Lys312***, Thr313

Note: The charged residues are in bold while the residues involved in the formation of H-bond are in italic.

### Identification of flexible residues in Cytoplasmic IκB proteins

To identify mobile structural elements, the atomic positional fluctuations for all IκB backbone atoms were monitored during the simulation time. A residue-based description of the local flexibility was obtained by calculating root mean square fluctuation (RMSF) values. The backbone RMSF values were calculated over the final 5 ns of the MD simulation. Residues that deviated more than 1 Å were considered to be highly flexible elements of the protein.

#### (i) IκBα

**Figure 9:**
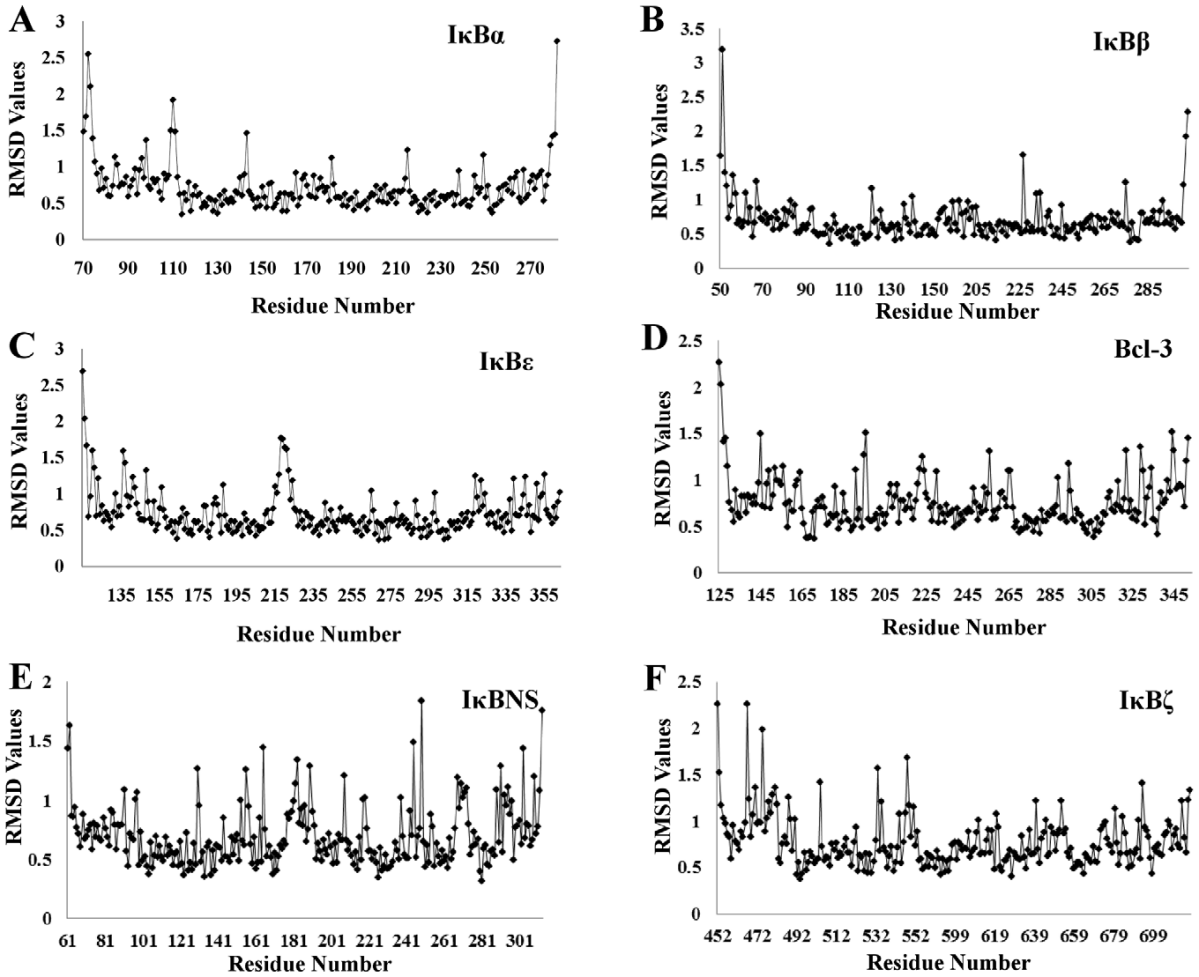
RMS deviations of individual amino acid residues of IκB proteins. A, B and C represent the results of the MD simulation for the cytoplasmic ARD domains of IκBα, IκBβ and IκBε, respectively, whereas D, E and F depict the RMSD fluctuation of the amino acid residues of nuclear ARD domains of Bcl-3, IκBNS and IκBζ during the MD simulation. In each case, amino acid residue numbers (actual) are plotted on the x-axis and RMS deviations (in Angstrom units) are plotted on the y-axis.

MD simulation studies have identified 32 amino acids with high RMSD fluctuations in IκBα. The most thermodynamically flexible residues in IκBα are: five residues (Asp73, Asp75, Glu85, Arg95 and Gln96) in ANK1; two (Asn109 and Leu110) in ANK2; four (Phe142, Arg143, Leu172 and His173) in ANK3; one (Cys215) in ANK5; and twenty (Trp258, Arg260, Pro261, Ser262, Thr263, Arg264, Gln267, Met279, Glu282, Ser283, Glu284, Asp285, Glu286, Glu287, Ser288, Tyr289, Asp290, Thr291, Glu292 and Ser293) in ANK6 along with its PEST motif (Figure 9A and S4A). Previous biochemical and crystallographic studies have shown crucial IκBα residues, which are liable to interact with p50/p65 heterodimer, thereby controlling the NF-κB subunits shuttling between the cytosol and nucleus [13], [14], [21], [51]. Please note that in our current study, we conducted the MD simulation of IκBα without its partners to identify whether or not the flexible residues mediate the interaction between IκBα and its partners. However, it is interesting to note that 19 out of the 32 most flexible residues, namely Asp73, Asp75, Glu85, Gln96, Asn109, Leu110, Arg143, Cys215, Trp258, Thr263, Gln267, Met279, Glu282, Glu284, Asp285, Glu286, Glu287, Ser288 and Tyr289, are considered to be essential for the interaction with p50/p65 subunits. Of these, Asp73, Asp75 and Glu85 of IκBα form a salt bridge with Lys301, Arg304 and Arg302 of the p65 NLS, respectively [13]. These salt bridges play a crucial role in masking the p65 NLS and thereby retaining the p50/p65 subunits in the cytoplasm. Apart from these interactions, Arg143, Cys215 and Trp258 form H-bonds with p65/p50 DD, further stabilizing the complex. Out of the 32 flexible residues, 12 encompass the PEST sequence, which plays an important role in direct electrostatic interactions with basic DNA binding loops, leading to dissociation of the p65 N-terminal end from DNA. Previous mutagenesis studies have shown that Glu282, Glu284, Asp285, Glu286 and Glu287 are considered to be important in mediating electrostatic interaction with p65 N-terminal domain [51] and are identified as highly flexible residues in our current study. These results suggest that IκBα is more flexible in finger loop regions, and this increased flexibility might play a crucial role in interactions with its partners. From the above results, we believe that our current approach is reliable and can be subsequently used for other IκB proteins.

#### (ii) IκBβ

RMSD analysis during the MD trajectory identified eighteen residues with large RMSD values in IκBβ. Of these, ten residues are mapped (Val50, Phe51, Gly52, Tyr53, Glu56, Asp57, Asp59, Ile67, Gln69 and Phe76) in ANK1, two residues (Tyr205 and Arg227) in ANK4, three residues (Arg275, Asp303 and Gly304) in ANK6 and one each in ANK2 (Leu121), ANK3 (His140) and ANK5 (Thr239) (Figure 9B and S4B). Since the crystal structure of the IκBβ-p65/p65 complex is known, interpreting our predicted results is straightforward. It is remarkable to note that 12 out of the 18 thermodynamically flexible residues, namely Gly52, Tyr53, Glu56, Asp57, Glu59, Ile67, Gln69, Phe76, Leu121, Tyr205, Thr239 and Arg275, are considered to be crucial residues in mediating the interaction with p65 homodimer [16]. Of those, IκBβ Glu59, Gln69 and Asp57 form H-bond with Lys301, Arg302 and 304 of the p65 NLS, respectively. Additionally, Glu56, Ile67 and Gln69 form van der Waals interactions with the p65 NLS. The IκBβ-p65/p65 complex is further stabilized by π-π stacking interactions between IκBβ Phe76 and p65 Phe318. Finally, Leu121, Tyr205, Thr239 and Arg275 of IκBβ interact with the p65 dimer interface, further stabilizing the complex. Previous studies reported that the IκBβ PEST sequence exhibits flexibility, and thus it probably is displaced from the DNA-protein interface [16]. The crystal complex used for the MD simulation studies did not contain the PEST motif; hence, we were not able to identify its flexibility. The reported crystal structure suggested that p65 NLS subunit A is tightly bound to IκBβ, whereas subunit B is exposed. This mode of binding is not likely to prevent the complex from entering the nucleus. Therefore, this structure is a representative of conformation adopted by the nuclear IκBβ-p65/p65 complex. However, the residues that did not participate in the above interactions might adopt different conformations in order to bind with p65/p65 dimer.

#### (iii) IκBε

RMSF calculations demonstrated that thirty one amino acid residues of IκBε are thermodynamically flexible. Of those residues, eleven are situated in ANK1 (Leu116, Thr117, Tyr118, Glu121, Asp122, Asp124, Ser127, Val138, Cys142, Leu143 and Glu149), nine in ANK3 (Gln189, Glu216, Pro217, Gly218, Arg219, Gln220, Leu221, Ser222 and His223), three in ANK6 (Leu299, Leu320 and Ala323), five in ANK7 (Leu340, Ser346, Glu352, Ser356 and Glu364) and one each in ANK2 (Leu157), ANK4 (Leu225) and ANK5 (Thr266). As expected, the finger loop regions of the model, especially between the ANK repeats (24 residues), were found to be responsible for the increasing fluctuations (Figure 9C and S4C). This flexibility mediates the binding of IκBε to its partners. It is noteworthy that 10 out of the 31 thermodynamically flexible residues, namely Asp122, Asp1124, Ser137, Val138, Glu149, Leu157, Gln189, Thr266, Ser346 and Ser356, present in our docking model interact with p50/p65 heterodimer. Similar studies have been conducted previously on other proteins such as Smad7, CYPB1 and IκBζ, whose identified thermodynamically flexible residues have been shown to interact with both substrates and receptors [21], [52], [53], further validating our docking model. It should also be noted that stimulus-induced cytoplasmic IκB degradation results in release of NF-κB to the nucleus, resulting in a transcriptional response that includes the IκB genes. The newly synthesized IκB protein enters into the nucleus and binds to NF-κB. Due to electrostatic repulsion, these complexes are brought back to the cytoplasm, thereby terminating the transcriptional process. During this process, IκB possesses different binding orientations. The remaining flexible residues that did not contribute in the above interactions might undertake a different binding orientation in order to bind with c-Rel and p65 containing NF-κB subunits [15], [54].

### Identification of flexible residues in nuclear IκB proteins

#### (i) Bcl-3

Bcl-3 contains thirty thermodynamically flexible residues. Of those 30 residues, nine are placed in ANK1 (Glu125, Asp126, Gly127, Asp128, Thr129, Arg145, Leu149, Gln152 and Glu156), six in ANK3 (Met191, Arg195, His196, Pro222, Gly223 and Thr224), three in ANK5 (Ile265, Lys266 and Gln289), seven in ANK7 (Leu3229, Lys330, Asn334, Arg344, Arg345, Arg351 and Gly352), one in ANK2 (Arg164) and two each in ANK4 (Arg230 and Arg256) and ANK6 (Val294, Arg322) (Figure 9D and S4D). It is interesting to note that 13 out of the 30 flexible residues, namely Met191, Arg230, Arg256, Ile265, Lys266, Gln289, Val294, Arg322, Leu329, Lys330, Asn334, Arg351 and Gly352, are considered to be crucial residues in the interaction with p50/p50 homodimer (Figure 7 and 8) and thereby in the transfer of the TAD to p50/p50 subunits. Biochemical studies have shown that Bcl-3 possesses diverse functions when in a complex with DNA-bound p50 homodimer [2]. This leads to different possible conformations of Bcl-3, which is in agreement with our current docking studies (Figure 7 and 8). Residues that did not contribute in these above interactions might be involved in those different conformations.

#### (ii) IκBNS

Thirty thermodynamically flexible residues were identified in Bcl-3. Of those residues, three are positioned in ANK1 (Glu61, Glu62 and Gln91), five in ANK4 (Phe165, Met182, Leu183, Arg190 and Gln208), four in ANK5 (Ile218, Lys219, Gln238 and Arg245), six in ANK6 (Arg249, Leu268, Pro270, Gly271, Pro272 and Pro273), eight in ANK7 (Thr289, Arg291, Leu293, Asn295, Arg302, Glu303, Arg312 and Gln313) and two each in ANK2 (His97 and Lys98) and ANK3 (His130 and Ile156) (Figure 9E and S4E). Of those 30 flexible residues, 11 are considered to be important for interactions with p50/p50 homodimer (Figure 6) and thereby in the negative regulation of TLR signaling. Previous studies have shown that IκBNS-deficient T cells are characterized by decreased IL-2 production, suggesting the positive regulation of IκBNS in a complex with DNA-bound p50/p50 homodimer [55]. It should be noted that due to the lack of a TAD in IκBNS, its positive regulation might be similar to complex B. Like other nuclear IκB proteins that exhibit dual functions when bound to their partners, IκBNS might also possess different conformations and play dual roles in the transcriptional process. The residues that did not contribute in the above binding might play crucial roles into the formation of different conformations. Additionally, IκBNS-mediated positive and negative regulation of NF-κB gene expression might depend upon the cell type expressing these genes.

#### (iii) IκBζ

Forty-seven flexible residues were identified in IκBζ. Of those residues, eighteen are located in ANK1 (Gly452, Asp453, Gly454, Asp455, Arg466, Arg467, Ala468, Tyr471, Val472, Leu473, Ala474, Arg475, Met477, Asn478, Ala479, Leu480, His481 and Met482), four in ANK2 (Lys486, Asn489, Gln491 and Leu504), three in ANK5 (Cys618, Arg620 and Lys621), eight in ANK6 (Leu645, Cys648, Phe651, Asn653, Thr674, Gln675, Arg680 and Arg684) and seven each in ANK3 (Cys521, Lys533, Lys544, Val547, Arg548, Ser549 and Gln551) and ANK7 (Arg692, Leu694, Pro707, Val708, Arg714, Lys717 and Gly718) (Figure 9F and S4F). The total number of flexible residues identified in our current study is higher compared to our previous studies, which is mainly due to the variation in methods [21]. It is remarkable to note that eight out of the 47 most flexible residues, namely Met477, Leu480, His481, Met482, Lys486, Arg548, Glu644 and Cys648, are known to important in mediating IκBζ-p50/p65 complex formation and thereby in inhibiting the transcriptional process. Additionally, nine residues out of the 47, namely Lys618, Arg620, Lys621, Cys648, Phe651, Asn653, Arg692, Lys717 and Gly718, are considered to be crucial for IκBζ-p50/p50 complex formation and thus in activating the transcriptional response [21]. The remaining thirty amino acids that did not participate in the above two interactions might interact with Brg1, CEBP1 STAT3 and p50/p65-CEBP, thereby regulating their function [22], [23], [24], [25], [26], [56].

### Prime factor for functional divergences in IκB proteins

The current studies have found that IκBα, IκBε, IκBNS and Bcl-3 possess approximately an equal number of flexible residues (30 amino acids), all of which are predominantly located in the finger loop region. IκBβ possesses a lower number of flexible residues compared to other IκB proteins. This is mainly due to the lack of a PEST motif in the structure; a corresponding motif in IκBα alone possesses 12 flexible residues. These cytoplasmic IκB proteins are structurally similar, and also the identified flexible residues are equal in number. However, of these identified flexible residues, only five (Asp73, Asp75, Gln96, Tyr181 and Cys215) corresponding to IκBα in Figure 1 are conserved. Here, we did not take into account residue conservation based on amino acid physico-chemical properties; rather, flexible residual position corresponding to IκBα was considered. In addition to the electrostatic surface of these IκB proteins, variation in flexible residual position might be the principal factor for the binding specificities with different combinations of NF-κB dimer. Nuclear IκB protein (IκBNS and Bcl-3) binds only p50/p50 homodimer and also possesses the same number of flexible residues. In this case, different conformations of IκB binding to p50/p50 dimer are possible, which can be inferred by our docking studies. These flexible residues might play a crucial factor in facilitating different conformations of interactions, thereby regulating the transcriptional response. Among these IκB proteins, IκBζ possesses a higher number of flexible residues (47 amino acid) compared to the IκB family. This increased flexibility might promote an interaction with NF-κB dimer as well as with other nuclear proteins, thereby regulating numerous transcriptional functions [5], [22], [24], [25], [26], [28], [56].

In conclusion, we have observed that the highly flexible finger loop portion contributes to major interactions with NF-κB. Nevertheless, from crystal structures and modeling studies, we observed conspicuous variations between cytoplasmic and nuclear IκB proteins bound to NF-κB subunits. The finger loop regions of cytoplasmic IκB proteins are on the left hand side, pointing towards the DD domain of NF-κB dimer (Figure 5). Conversely, the finger loop regions of nuclear IκB proteins are positioned on the right hand side and project away from the DD of NF-κB dimer (Figure 6, 7 and 8).

Taken together, we have elucidated models of IκB proteins, carried out detailed MD simulation and identified thermodynamically flexible residues. These thermodynamically flexible residues are considered to be the key factors responsible for the exhibited binding specificities with different combinations of NF-κB dimer. Moreover, the docking studies allowed us to understand the positive and negative regulation of IκB protein binding with different NF-κB subunits in the context of TLR signaling. Finally, our current models can be utilized as a guide for future experimental and computational studies. The presented modeling approach can also be extended to other repetitive protein domains.

## Supporting Information

Figure S1 **Surface electrostatic representation of ARD.** A, B and C show the calculated electrostatic surface of the cytoplasmic ANK repeat domain with blue-colored regions depicting positively charged basic patches and red-colored regions depicting negatively charged acidic patches. The negatively charged acidic C-terminal PEST motif, which is known to be involved in electrostatic repulsion, is circled in dotted lines. C, D and E possess both positively and negatively charged surfaces at their C-termini, which are marked in circled and dotted circled lines, respectively.
(TIF)

Figure S2 **Superimposition of initial structure with the final snapshot obtained from MD simulation studies.** Differences between the final snapshots of (A) IκBα (yellow), (B) IκBβ (aquamarine), (C) IκBε (sky blue), (D) Bcl-3 (khaki), (E) IκBNS (orchid) and (F) IκBζ (light green) and their respective initial structures (salmon). Variations are mainly observed within the residue-joining ANK repeats and also between ANK repeats, which are represented as dots and asterisks, respectively.
(TIF)

Figure S3 **Benchmark of docking programs using known crystal structure complexes.** The p50/p65 hetero- and p65/p65 homodimers, represented as ribbon diagrams, are shown in blue and green as well as pink and violet, respectively. The native IκB protein poses are red colored, and the IκB protein poses predicted by the docking programs are in yellow (ZDOCK) and dark blue (GRAMM-X), respectively. (**A**) Representation of the IκBα-p50/p65 complex and (**B**) IκBβ-p65/p65 homodimer complex.
(TIF)

Figure S4 **Molecular models of ARD domains.** Crystal structures of the ARD domains of IκBα (A), IκBβ (B) and Bcl-3 (C) are colored in yellow, aquamarine and khaki, respectively. Homology models of the ARD domains of IκBε (D), IκBNS (E) and IκBζ (F) are colored in sky blue, orchid and light green, respectively. The positions of the flexible residues determined by the molecular dynamic simulations are highlighted in red.
(TIF)

Tables S1 (A) IκBε-p50/p65 heterodimer. (B) IκBNS-p50/p50 homodimer. (C) Bcl3-p50/p50 homodimer.
(DOC)

## References

[1] Hoffmann A, Baltimore D (2006). Circuitry of nuclear factor kappaB signaling.. Immunol Rev.

[2] Ghosh S, Hayden MS (2008). New regulators of NF-kappaB in inflammation.. Nat Rev Immunol.

[3] Hayden MS, Ghosh S (2008). Shared principles in NF-kappaB signaling.. Cell.

[4] Michel F, Soler-Lopez M, Petosa C, Cramer P, Siebenlist U (2001). Crystal structure of the ankyrin repeat domain of Bcl-3: a unique member of the IkappaB protein family.. EMBO J.

[5] Yamamoto M, Takeda K (2008). Role of nuclear IkappaB proteins in the regulation of host immune responses.. J Infect Chemother.

[6] Agrawal AK, Hop CE, Pang J, Silva Elipe MV, Desai RC (2005). In vitro metabolism of a new oxazolidinedione hypoglycemic agent utilizing liver microsomes and recombinant human cytochrome P450 enzymes.. J Pharm Biomed Anal.

[7] Sedgwick SG, Smerdon SJ (1999). The ankyrin repeat: a diversity of interactions on a common structural framework.. Trends Biochem Sci.

[8] Mosavi LK, Cammett TJ, Desrosiers DC, Peng ZY (2004). The ankyrin repeat as molecular architecture for protein recognition.. Protein Sci.

[9] Forrer P, Stumpp MT, Binz HK, Pluckthun A (2003). A novel strategy to design binding molecules harnessing the modular nature of repeat proteins.. FEBS Lett.

[10] Main ER, Jackson SE, Regan L (2003). The folding and design of repeat proteins: reaching a consensus.. Curr Opin Struct Biol.

[11] Malek S, Huxford T, Ghosh G (1998). Ikappa Balpha functions through direct contacts with the nuclear localization signals and the DNA binding sequences of NF-kappaB.. J Biol Chem.

[12] Ghosh S, May MJ, Kopp EB (1998). NF-kappa B and Rel proteins: evolutionarily conserved mediators of immune responses.. Annu Rev Immunol.

[13] Jacobs MD, Harrison SC (1998). Structure of an IkappaBalpha/NF-kappaB complex.. Cell.

[14] Huxford T, Huang DB, Malek S, Ghosh G (1998). The crystal structure of the IkappaBalpha/NF-kappaB complex reveals mechanisms of NF-kappaB inactivation.. Cell.

[15] Li Z, Nabel GJ (1997). A new member of the I kappaB protein family, I kappaB epsilon, inhibits RelA (p65)-mediated NF-kappaB transcription.. Mol Cell Biol.

[16] Malek S, Huang DB, Huxford T, Ghosh S, Ghosh G (2003). X-ray crystal structure of an IkappaBbeta x NF-kappaB p65 homodimer complex.. J Biol Chem.

[17] Hertlein E, Wang J, Ladner KJ, Bakkar N, Guttridge DC (2005). RelA/p65 regulation of IkappaBbeta.. Mol Cell Biol.

[18] Hoffmann A, Natoli G, Ghosh G (2006). Transcriptional regulation via the NF-kappaB signaling module.. Oncogene.

[19] Kuwata H, Matsumoto M, Atarashi K, Morishita H, Hirotani T (2006). IkappaBNS inhibits induction of a subset of Toll-like receptor-dependent genes and limits inflammation.. Immunity.

[20] Wessells J, Baer M, Young HA, Claudio E, Brown K (2004). BCL-3 and NF-kappaB p50 attenuate lipopolysaccharide-induced inflammatory responses in macrophages.. J Biol Chem.

[21] Manavalan B, Govindaraj RG, Lee G, Choi S Molecular Modeling-based Evaluation of Dual Function of IκBζ Ankyrin Repeat Domain in Toll-like Receptor Signaling.. J Mol Recognit.

[22] Matsuo S, Yamazaki S, Takeshige K, Muta T (2007). Crucial roles of binding sites for NF-kappaB and C/EBPs in IkappaB-zeta-mediated transcriptional activation.. Biochem J.

[23] Motoyama M, Yamazaki S, Eto-Kimura A, Takeshige K, Muta T (2005). Positive and negative regulation of nuclear factor-kappaB-mediated transcription by IkappaB-zeta, an inducible nuclear protein.. J Biol Chem.

[24] Totzke G, Essmann F, Pohlmann S, Lindenblatt C, Janicke RU (2006). A novel member of the IkappaB family, human IkappaB-zeta, inhibits transactivation of p65 and its DNA binding.. J Biol Chem.

[25] Trinh DV, Zhu N, Farhang G, Kim BJ, Huxford T (2008). The nuclear I kappaB protein I kappaB zeta specifically binds NF-kappaB p50 homodimers and forms a ternary complex on kappaB DNA.. J Mol Biol.

[26] Wu Z, Zhang X, Yang J, Wu G, Zhang Y (2009). Nuclear protein IkappaB-zeta inhibits the activity of STAT3.. Biochem Biophys Res Commun.

[27] Wheeler DL, Barrett T, Benson DA, Bryant SH, Canese K (2008). Database resources of the National Center for Biotechnology Information.. Nucleic Acids Res.

[28] Yamazaki S, Muta T, Takeshige K (2001). A novel IkappaB protein, IkappaB-zeta, induced by proinflammatory stimuli, negatively regulates nuclear factor-kappaB in the nuclei.. J Biol Chem.

[29] Eswar N, Webb B, Marti-Renom MA, Madhusudhan MS, Eramian D (2006). Comparative protein structure modeling using Modeller.. Curr Protoc Bioinformatics Chapter.

[30] Edgar RC (2004). MUSCLE: multiple sequence alignment with high accuracy and high throughput.. Nucleic Acids Res.

[31] Jo S, Kim T, Iyer VG, Im W (2008). CHARMM-GUI: a web-based graphical user interface for CHARMM.. J Comput Chem.

[32] Krieger E, Darden T, Nabuurs SB, Finkelstein A, Vriend G (2004). Making optimal use of empirical energy functions: force-field parameterization in crystal space.. Proteins.

[33] Wallner B, Fang H, Elofsson A (2003). Automatic consensus-based fold recognition using Pcons, ProQ, and Pmodeller.. Proteins.

[34] Ponder JW, Case DA (2003). Force fields for protein simulations.. Adv Protein Chem.

[35] McGuffin LJ (2008). The ModFOLD server for the quality assessment of protein structural models.. Bioinformatics.

[36] Pawlowski M, Gajda MJ, Matlak R, Bujnicki JM (2008). MetaMQAP: a meta-server for the quality assessment of protein models.. BMC Bioinformatics.

[37] Maiti R, Van Domselaar GH, Zhang H, Wishart DS (2004). SuperPose: a simple server for sophisticated structural superposition.. Nucleic Acids Res.

[38] W.L. Da The PyMOL Molecular Graphics System (2002). DeLano Scientific.

[39] Tovchigrechko A, Vakser IA (2006). GRAMM-X public web server for protein-protein docking.. Nucleic Acids Res.

[40] Chen R, Li L, Weng Z (2003). ZDOCK: an initial-stage protein-docking algorithm.. Proteins.

[41] Ghosh G, van Duyne G, Ghosh S, Sigler PB (1995). Structure of NF-kappa B p50 homodimer bound to a kappa B site.. Nature.

[42] Muller CW, Rey FA, Sodeoka M, Verdine GL, Harrison SC (1995). Structure of the NF-kappa B p50 homodimer bound to DNA.. Nature.

[43] Reynolds C, Damerell D, Jones S (2009). ProtorP: a protein-protein interaction analysis server.. Bioinformatics.

[44] Cole C, Barber JD, Barton GJ (2008). The Jpred 3 secondary structure prediction server.. Nucleic Acids Res.

[45] Mizuguchi K, Deane CM, Blundell TL, Johnson MS, Overington JP (1998). JOY: protein sequence-structure representation and analysis.. Bioinformatics.

[46] Huxford T, Mishler D, Phelps CB, Huang DB, Sengchanthalangsy LL (2002). Solvent exposed non-contacting amino acids play a critical role in NF-kappaB/IkappaBalpha complex formation.. J Mol Biol.

[47] Hirotani T, Lee PY, Kuwata H, Yamamoto M, Matsumoto M (2005). The nuclear IkappaB protein IkappaBNS selectively inhibits lipopolysaccharide-induced IL-6 production in macrophages of the colonic lamina propria.. J Immunol.

[48] Nolan GP, Fujita T, Bhatia K, Huppi C, Liou HC (1993). The bcl-3 proto-oncogene encodes a nuclear I kappa B-like molecule that preferentially interacts with NF-kappa B p50 and p52 in a phosphorylation-dependent manner.. Mol Cell Biol.

[49] Bundy DL, McKeithan TW (1997). Diverse effects of BCL3 phosphorylation on its modulation of NF-kappaB p52 homodimer binding to DNA.. J Biol Chem.

[50] Caamano JH, Perez P, Lira SA, Bravo R (1996). Constitutive expression of Bc1-3 in thymocytes increases the DNA binding of NF-kappaB1 (p50) homodimers in vivo.. Mol Cell Biol.

[51] Sue SC, Dyson HJ (2009). Interaction of the IkappaBalpha C-terminal PEST sequence with NF-kappaB: insights into the inhibition of NF-kappaB DNA binding by IkappaBalpha.. J Mol Biol.

[52] Hariharan R, Pillai MR (2008). Structure-function relationship of inhibitory Smads: Structural flexibility contributes to functional divergence.. Proteins.

[53] Li X, Baudry J, Berenbaum MR, Schuler MA (2004). Structural and functional divergence of insect CYP6B proteins: From specialist to generalist cytochrome P450.. Proc Natl Acad Sci U S A.

[54] Kearns JD, Basak S, Werner SL, Huang CS, Hoffmann A (2006). IkappaBepsilon provides negative feedback to control NF-kappaB oscillations, signaling dynamics, and inflammatory gene expression.. J Cell Biol.

[55] Touma M, Antonini V, Kumar M, Osborn SL, Bobenchik AM (2007). Functional role for I kappa BNS in T cell cytokine regulation as revealed by targeted gene disruption.. J Immunol.

[56] Yamazaki S, Matsuo S, Muta T, Yamamoto M, Akira S (2008). Gene-specific requirement of a nuclear protein, IkappaB-zeta, for promoter association of inflammatory transcription regulators.. J Biol Chem.

