# Supplementary figures and images for: Structure-Function Relationship of Cytoplasmic and Nuclear IκB Proteins: An In Silico Analysis
Source: PLoS One. 2010 Dec 23;5(12):e15782. doi: 10.1371/journal.pone.0015782 (PMC3009747; doi:10.1371/journal.pone.0015782)

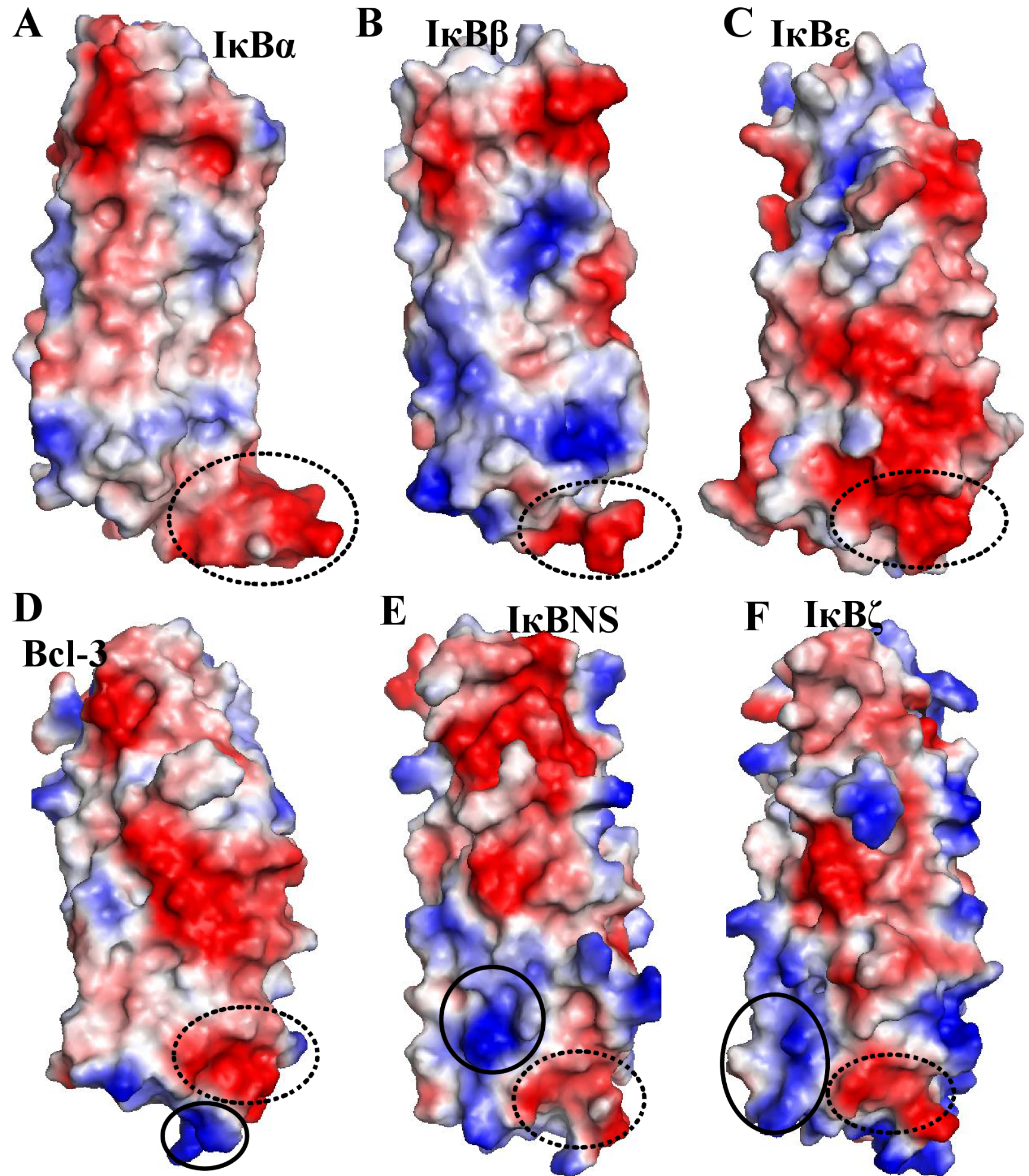

Supplement: Figure S1 — Surface electrostatic representation of ARD. A, B and C show the calculated electrostatic surface of the cytoplasmic ANK repeat domain with blue-colored regions depicting positively charged basic patches and red-colored regions depicting negatively charged acidic patches. The negatively charged acidic C-terminal PEST motif, which is known to be involved in electrostatic repulsion, is circled in dotted lines. C, D and E possess both positively and negatively charged surfaces at their C-termini, which are marked in circled and dotted circled lines, respectively. (TIF) [file pone.0015782.s001.tif]

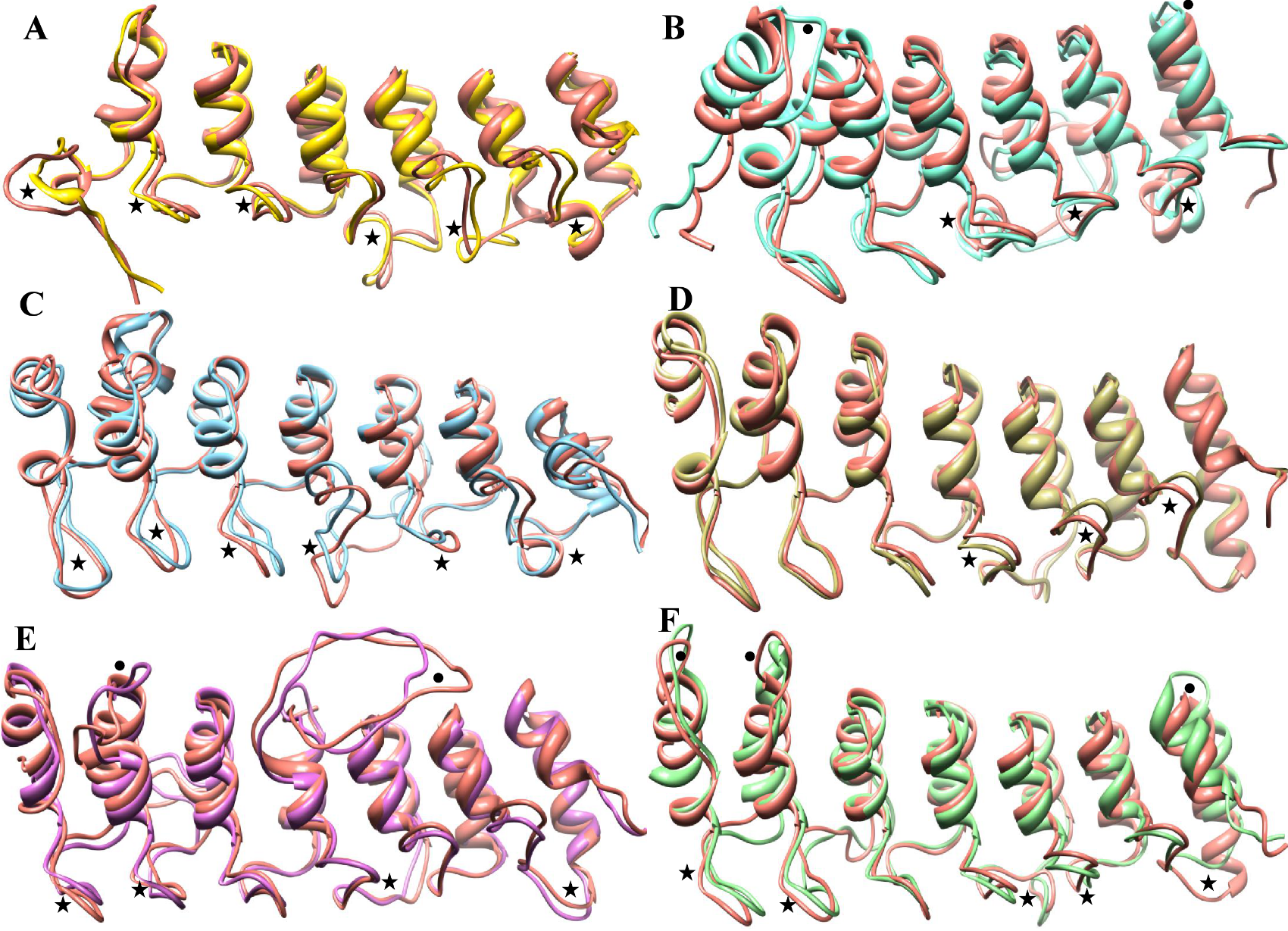

Supplement: Figure S2 — Superimposition of initial structure with the final snapshot obtained from MD simulation studies. Differences between the final snapshots of (A) IκBα (yellow), (B) IκBβ (aquamarine), (C) IκBε (sky blue), (D) Bcl-3 (khaki), (E) IκBNS (orchid) and (F) IκBζ (light green) and their respective initial structures (salmon). Variations are mainly observed within the residue-joining ANK repeats and also between ANK repeats, which are represented as dots and asterisks, respectively. (TIF) [file pone.0015782.s002.tif]

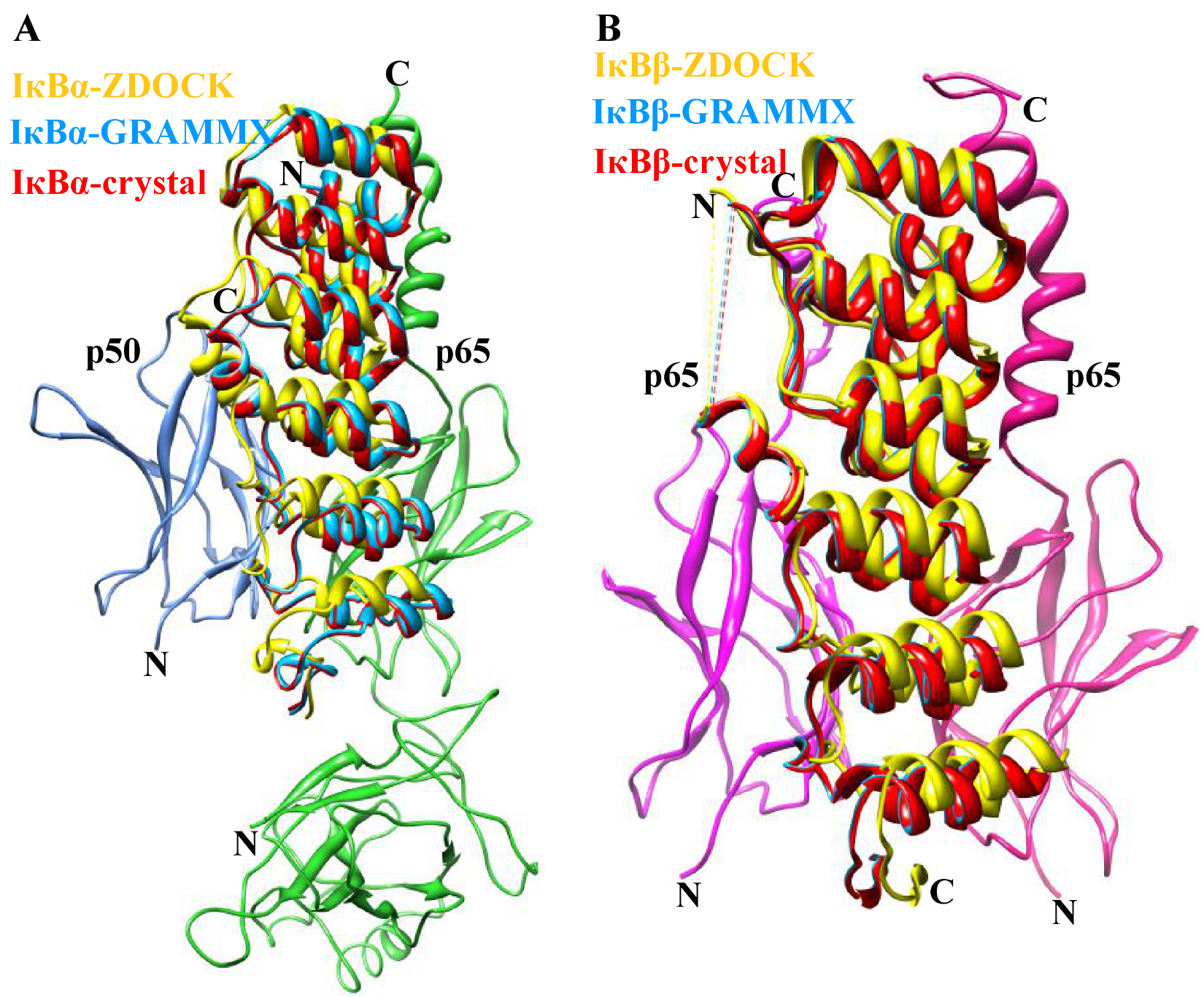

Supplement: Figure S3 — Benchmark of docking programs using known crystal structure complexes. The p50/p65 hetero- and p65/p65 homodimers, represented as ribbon diagrams, are shown in blue and green as well as pink and violet, respectively. The native IκB protein poses are red colored, and the IκB protein poses predicted by the docking programs are in yellow (ZDOCK) and dark blue (GRAMM-X), respectively. (A) Representation of the IκBα-p50/p65 complex and (B) IκBβ-p65/p65 homodimer complex. (TIF) [file pone.0015782.s003.tif]

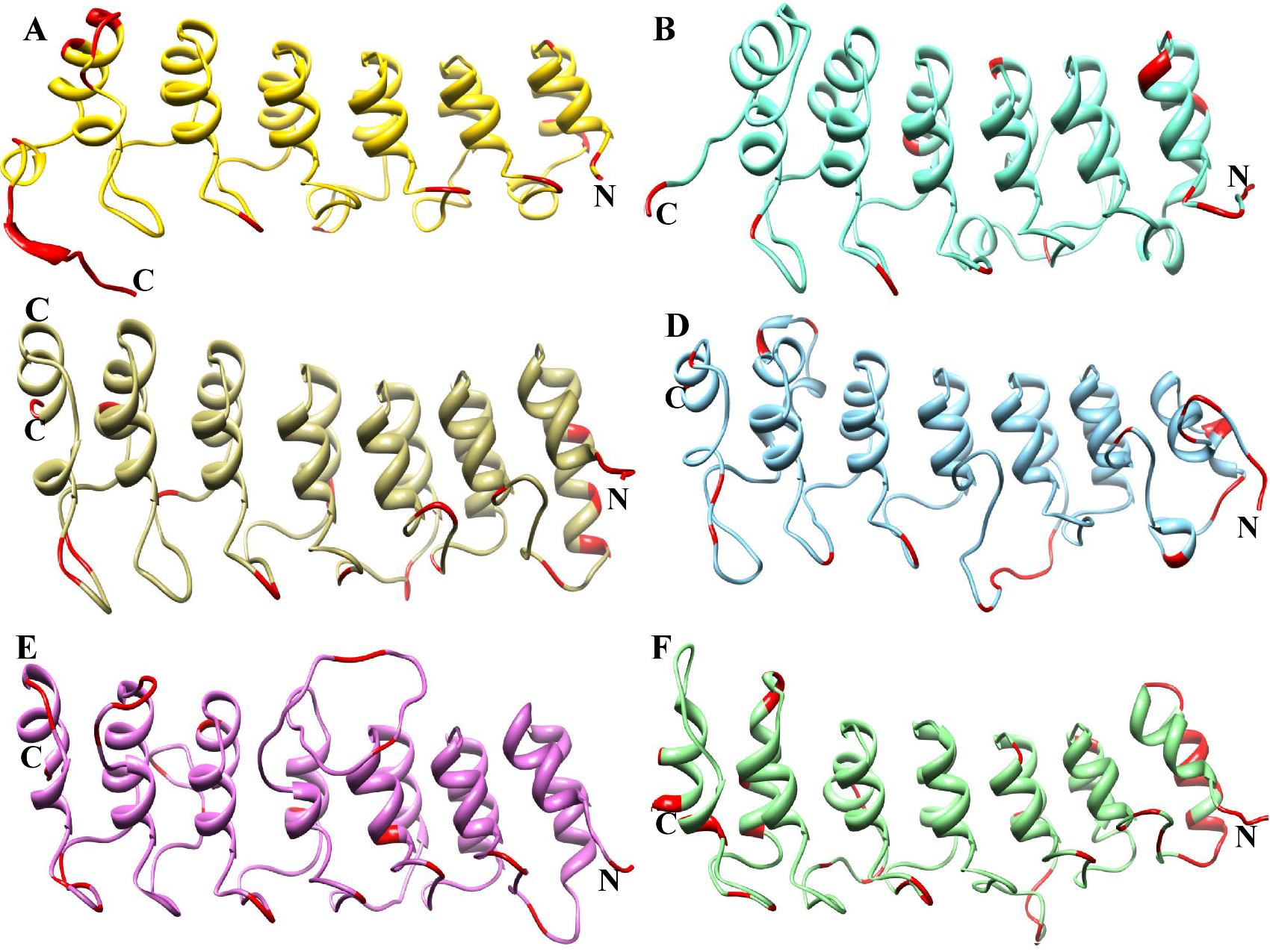

Supplement: Figure S4 — Molecular models of ARD domains. Crystal structures of the ARD domains of IκBα (A), IκBβ (B) and Bcl-3 (C) are colored in yellow, aquamarine and khaki, respectively. Homology models of the ARD domains of IκBε (D), IκBNS (E) and IκBζ (F) are colored in sky blue, orchid and light green, respectively. The positions of the flexible residues determined by the molecular dynamic simulations are highlighted in red. (TIF) [file pone.0015782.s004.tif]
