# Supplementary material for: Structure-Function Relationship of Cytoplasmic and Nuclear IκB Proteins: An In Silico Analysis
Source: PLoS One. 2010 Dec 23;5(12):e15782. doi: 10.1371/journal.pone.0015782 (PMC3009747; doi:10.1371/journal.pone.0015782)
Supplement: Tables S1 — (A) IκBε-p50/p65 heterodimer. (B) IκBNS-p50/p50 homodimer. (C) Bcl3-p50/p50 homodimer. (DOC) [file pone.0015782.s005.doc]

**Tables S1.**

(A) IκBε-p50/p65 heterodimer.

| **Complex Rankings** | **List of interacting residues in IκBε** | **Number of H-bonds** | **Number of salt bridges** | **Interface area** |
| --- | --- | --- | --- | --- |
| **Complex 4** | D122, D124, L126, L129, A130, I132, H133, A135, A135, S137, V138, N155, N156, L157, Y158, Q159, N159, L164, H167, L168, D169, Q189, H190, N203, R209, W2332, T266, I355, S356 | 7 | 5 | 1360 |
| **Complex 7** | E134, C141, C142, A144, F145, P147, Q170, P171, D172, R175, L179, K180, G181, S183, I185, L186, L204, A205, C208, E212, E216, P217, G218, R219, Q220, P248, E251, L252, L254, Q255,N256, G257, D259, Q289, A290, G291, V332, E333, Q338, D364 | 7 | 2 | 1808 |
| **Complex 13** | E121, D122, L157, H190, R200, L225, D226, L227, K230, W232, Q233, G234, D261, V262, Q263, G265, T266, S267, K269, E277, R293, R297, M298, L299, N300, C302, R311, E342, L344 | 8 | 3 | 1533 |
| **Complex 17** | Y118, S120, E121, D122, D124, L129, N156, L157, Y158, Q159, L164, H167, L168, H190, D192, R200, R201, W232, Q233, L243, R245, K269, E277, T278, Q279, G310, R311, L340, L344, L345, S346, Y347, D351, D352, K354 | 6 | 5 | 1637 |
| **Complex 26** | N314, S315, L320, C321, E322, A323, N331, V332, D334, E335, T336, D337, Q338, L348, F350, D351, L353, I355, S356, G357, K358, P359, L360, L361, T363, D364, | 8 | 3 | 1409 |

(B) IκBNS-p50/p50 homodimer.

| **Complex Rankings** | **List of interacting residues in IκBNS** | **Number of H-bonds** | **Number of salt bridges** | **Interface area** |
| --- | --- | --- | --- | --- |
| **Complex 2** | R90, Q91, I94, R95, D96, H97, G99, N126, T128, D129, H130, G132, F152, I156, V158, D159, L160, D161, R163, D164, F165, L207, Q208, M209, G210, S212, T214, I218, K219, S220, N221, R249, F251, H257, G285, D287, P288, T289, R291, N295, K302, Q313 | 10 | 2 | 1844 |
| **Complex 4** | Q91, E116, D117, S120, F152, K153, S154, I156, Q157, V158, D159, L160, E161, A162, R163, F165, L207, Q208, M209,G210, S212, H213, T214, I218, R245, D247, L248, R249, F251 | 6 | 4 | 1395 |
| **Complex 5** | Y81, Q91, E124, N126, T128, S154, I156, Q157, D159, E161, A162, F165, S212, T214, R245, D247, L248, R249, F251, M254, K255, A283, A284, Q313 | 11 | 5 | 1424 |
| **Complex 8** | M88, Y89, R90, Q91, R95, S120, G122, E124, N126, A127, T128, H130, I156, Q157, D159, R163, F165, I218, F251, N253, M254, K255, H257, K285, D287, T289, L290, R291, L293, N295 | 9 | 4 | 1706 |
| **Complex 9** | F70, R73, G74, L75, R76, W77, K100, A105, A108, Q111, L113, V138, T141, Y142, L178, N179, A190, A181, M182, L183, P184, S186, V187, C188, P189, R190, M191, S194, Q195, R199 | 11 | 1 | 1487 |

(C) Bcl3-p50/p50 homodimer.

| **Complex Rankings** | **List of interacting residues in Bcl3** | **Number of H-bonds** | **Number of salt bridges** | **Interface area** |
| --- | --- | --- | --- | --- |
| **Complex 45**  **(Complex A)** | M191, D226, E228, A229, R230, R252, D261, V263, I265, G268, L288, Q289, H290, G291, A292, N293, V294, N295, Q297, M298, Y299, G301, L320, V321, R322, S323, G324, A325, D326, S328, L329, K330, N331, C332, N334, R351, G352 | 9 | 2 | 1644 |
| **Complex 57**  **(Complex B)** | R230, L254, E255, R256, G257, D259, I260, D261, V263, D264, K266, G268, L288, Q289, H290, G291, A292, N293, Y299, S300, G301, D326, R351, G352 | 8 | 1 | 1216 |
| **Complex 63** | R141, T175, H207, R208, P210, T211, R214, E245, Q247, E248, Q251, N278, S282, Q289, R311, G312, L313, R342 | 11 | 4 | 1138 |
| **Complex 72** | S189, M191, L193, P222, T224, D226, D259, D261, V263, I265, Q289, H290, G291, N293, V294, N295, Q297, Y299, V321, R322, S323, G324, D326, L329, K330, C332, N334, R351, G352 | 5 | 2 | 1451 |
| **Complex 78** | Q151, Q152, G154, E156, T185, G187, S189, M191, A221, P222, G323, T224, D226, Q251, E255, R256, G257, I260, L288, Q289, H290, G291, V321, R322, S323, G324, G352 | 7 | 3 | 1508 |
